# Blockade of immune checkpoints in lymph nodes through locoregional delivery augments cancer immunotherapy

**DOI:** 10.1126/scitranslmed.aay3575

**Published:** 2020-09-30

**Authors:** David M. Francis, Margaret P. Manspeaker, Alex Schudel, Lauren F. Sestito, Meghan J. O’Melia, Haydn T. Kissick, Brian P. Pollack, Edmund K. Waller, Susan N. Thomas

**Affiliations:** 1 School of Chemical and Biomolecular Engineering, Georgia Institute of Technology, Atlanta, GA 30332, USA.; 2 Parker H. Petit Institute for Bioengineering and Bioscience, Georgia Institute of Technology, Atlanta, GA 30332, USA.; 3 School of Material Science and Engineering, Georgia Institute of Technology, Atlanta, GA 30332, USA.; 4 Wallace H. Coulter Department of Biomedical Engineering, Georgia Institute of Technology and Emory University, Atlanta, GA 30332, USA.; 5 Winship Cancer Institute, Emory University, Atlanta, GA 30322, USA.; 6 Department of Urology, Emory University School of Medicine, Atlanta, GA 30322, USA.; 7 Emory Vaccine Center, Emory University School of Medicine, Atlanta, GA 30322, USA.; 8 Atlanta Veterans Affairs Medical Center, Decatur, GA 30033, USA.; 9 Departments of Dermatology and Pathology and Laboratory Medicine, Emory University School of Medicine, Atlanta, GA 30322, USA.; 10 George W. Woodruff School of Mechanical Engineering, Georgia Institute of Technology, Atlanta, GA 30332, USA.

## Abstract

Systemic administration of immune checkpoint blockade (ICB) monoclonal antibodies (mAbs) can unleash antitumor functions of T cells but is associated with variable response rates and off-target toxicities. We hypothesized that antitumor efficacy of ICB is limited by the minimal accumulation of mAb within tissues where antitumor immunity is elicited and regulated, which include the tumor microenvironment (TME) and secondary lymphoid tissues. In contrast to systemic administration, intratumoral and intradermal routes of administration resulted in higher mAb accumulation within both the TME and its draining lymph nodes (LNs) or LNs alone, respectively. The use of either locoregional administration route resulted in pronounced T cell responses from the ICB therapy, which developed in the secondary lymphoid tissues and TME of treated mice. Targeted delivery of mAb to tumor-draining lymph nodes (TdLNs) alone was associated with enhanced antitumor immunity and improved therapeutic effects compared to conventional systemic ICB therapy, and these effects were sustained at reduced mAb doses and comparable to those achieved by intratumoral administration. These data suggest that locoregional routes of administration of ICB mAb can augment ICB therapy by improving immunomodulation within TdLNs.

## INTRODUCTION

Immune checkpoint blockade (ICB) using monoclonal antibodies (mAbs) specific to cytotoxic T lymphocyte antigen 4 (CTLA-4) and to programmed cell death 1 (PD-1) or its ligands has emerged as one of the most promising approaches in cancer immunotherapy to invigorate antitumor immunity (1, 2). CTLA-4 is a transmembrane receptor found constitutively on regulatory T cells (T_regs_) and is limited in its expression by CD4 and CD8 T cells immediately after engagement of the T cell receptor. CTLA-4 directly competes with CD28 for B7 ligand binding on antigen-presenting cells (APCs), consequently leading to T cell anergy (3). Similarly, surface expression of PD-1 is broadly induced after T cell activation, and PD-1 is thought to function in peripheral tissues through its binding interactions with PD-1 ligands (PD-L1 and PD-L2) found on many cell subtypes including predominantly, but not limited to, tumor cells and APCs, respectively. After PD-1:ligand engagement, T cell function is dampened—an effect that protects the host during viral infection from immune-mediated tissue destruction leading to T cell exhaustion (3). By blocking these inhibitory pathways using function-blocking mAbs, activation and cytotoxic capabilities of T cells can be restored (1, 3).

Although the canonical view on ICB therapy effects is that they are mediated primarily within the tumor microenvironment (TME) by restoring antitumor functions of infiltrating T cells, evidence of the pleotropic effects of ICB mAbs continues to amass. Specific isotypes of anti-CTLA-4 (aCTLA-4) mAbs [immunoglobulin G2a (IgG2a)] mediate the depletion of tumor-resident T_regs_ (trT_regs_) via antibody-dependent cellular cytotoxicity, although other isotypes (IgG1) do not (4–6). In addition, whereas anti–PD-1 (aPD-1) mAb has been shown to restore the effector functions of CD8 and CD4 T cells (7), CD28 stimulation is required for aPD-1 efficacy, suggesting a role of B7-expressing APCs (8). aPD-1 has also been shown to modulate a stem-like CD8 T cell population capable of proliferating and giving rise to T cells of a tumor-killing effector-like phenotype (9–11). Furthermore, PD-L1 expression on tumor cells is not required for disease progression and aPD-1 efficacy in certain cancer types (12–14). Both aCTLA-4 and aPD-1 therapy have also been shown to broaden the repertoire of tumor-specific CD8 T cell clones (15–17), which is associated with improved clinical outcomes (18, 19). Solely blocking checkpoint pathways in the TME may thus not be sufficient to generate high response rates after ICB therapy.

To this end, appreciation for lymphoid tissues as critical in the generation of effective immunotherapy responses is increasing (20, 21). CD103^+^ APCs transport antigen to tumor-draining lymph nodes (TdLNs) where they can prime naïve CD8 T cells (22, 23). Moreover, TdLNs are involved in mediating the effects of aCTLA-4 (24) and aPD-1 therapy (25). The presence of the aforementioned stem-like CD8 T cell compartment has been observed in mouse and human LNs, in addition to the TME, suggesting these tissues as a potential source of tumor-infiltrating lymphocytes (TILs) (26). However, the TME and TdLNs are poorly accessed using systemic drug administration (27–29), the predominant route used in both preclinical tumor models and human patients, which may limit drug effects. Clinical studies have reported dose-efficacy relationships of aCTLA-4 and aPD-1 therapies (30, 31). Increasing the availability of ICB mAb within target tissues, including the TME and lymphoid tissues that are enriched in tumor-specific T cells, thus has the potential to improve ICB therapy.

Previous reports have described improvements in antitumor responses using intratumoral (i.t.) administration routes compared to traditional systemic administration (32–34). However, less is known about the antitumor effects of ICB modulation in LNs, although peri-tumoral administration has previously been investigated (24, 35) and subcutaneous (s.c.) administration is being explored in the clinic (36). Note that mAbs are large molecules (150 kDa) and thus are transported differently than traditional small-molecule drugs or other smaller biologics. Specifically, injection of compounds similarly sized to mAbs into the interstitium of peripheral tissues results in clearance from the injection site via the initial lymphatics and thus accumulation of such compounds in draining LNs (37). We hypothesized that mAbs would behave similarly, and therefore, direct administration into peripheral tissues would improve LN delivery of mAbs, allowing for improvement of ICB therapeutic effects. Our results in three preclinical solid tumor models (using melanoma and breast cancer cell lines) support the hypothesis that modulation of immune checkpoint pathways in (Td)LNs using locoregional administration of ICB mAbs enhances anti-tumor efficacy, enables dose sparing, and has the potential to reduce treatment-induced toxicity compared to systemically administered therapy.

## RESULTS

### Tumor-directed ICB augments local therapeutic responses

**Fig. 1.**
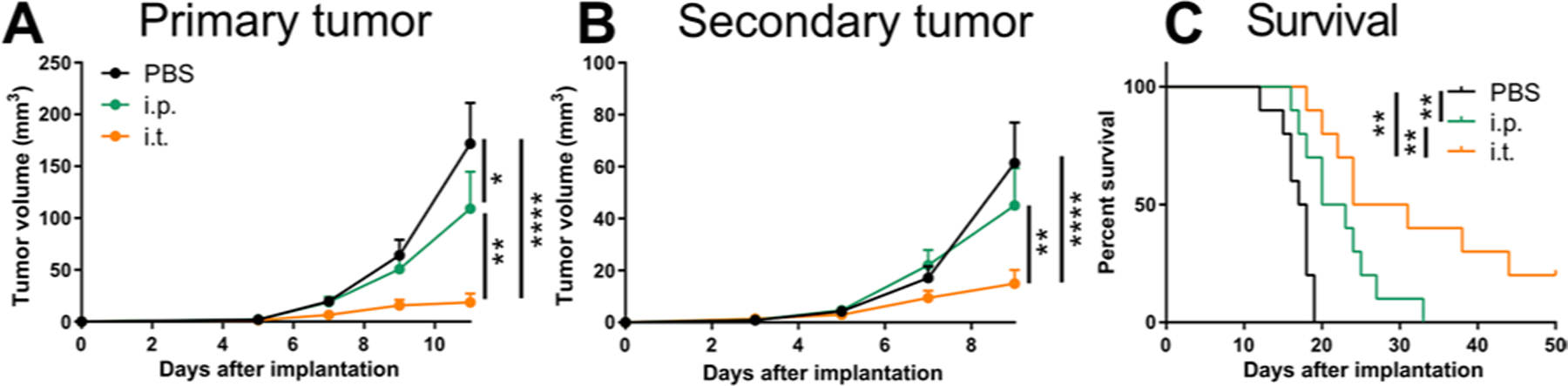
Intratumoral administration of ICB promotes systemic antitumor immunity. After B16F10 implantation, mice were administered 150 μg each of aPD-1 and aCTLA-4 (9H10) mAb on days 5, 7, and 9 after tumor implantation i.p. or i.t. (**A**) Tumor growth curve of primary tumor (day 0 tumor implant). (**B**) Tumor growth curve of secondary tumor (day 2 tumor implant, nontreated tumor). (**C**) Survival curves of mice. Combined data of two independent repeats (total *n* = 10). Statistical analyses were done using ANOVA with Tukey’s test. Log-rank (Mantel-Cox) test for survival curves. *P < 0.05, **P < 0.01, and ****P < 0.0001. Data are represented as means + SEM.

To evaluate whether augmenting the accumulation of administered mAb drug within target tissues can improve the effects of ICB, survival studies were performed in a poorly ICB-responsive tumor model (B16F10 melanoma) using both aCTLA-4 and aPD-1 mAbs and comparing intraperitoneal (i.p.) versus i.t. routes of administration. Modest reductions in tumor growth were induced by i.p. administration of ICB mAb; however i.t. administration instead resulted in profound reductions in tumor growth (Fig. 1A). To explore the potential effects on priming and expansion of T cells in response to endogenous tumor antigen, the systemic response of untreated tumors in the contralateral (c.l.) dorsal skin was concurrently monitored. Untreated tumors were found to be reduced in animals treated i.t. with ICB therapy (Fig. 1B). The net effect was a prolongation of mouse survival with i.t. compared to i.p. administration of ICB mAb (Fig. 1C). These data demonstrate the capacity of tumor-localized ICB therapy to elicit a systemically functional antitumor immune response that exceeds the effects of systemically administered ICB therapy.

**Fig. 2.**
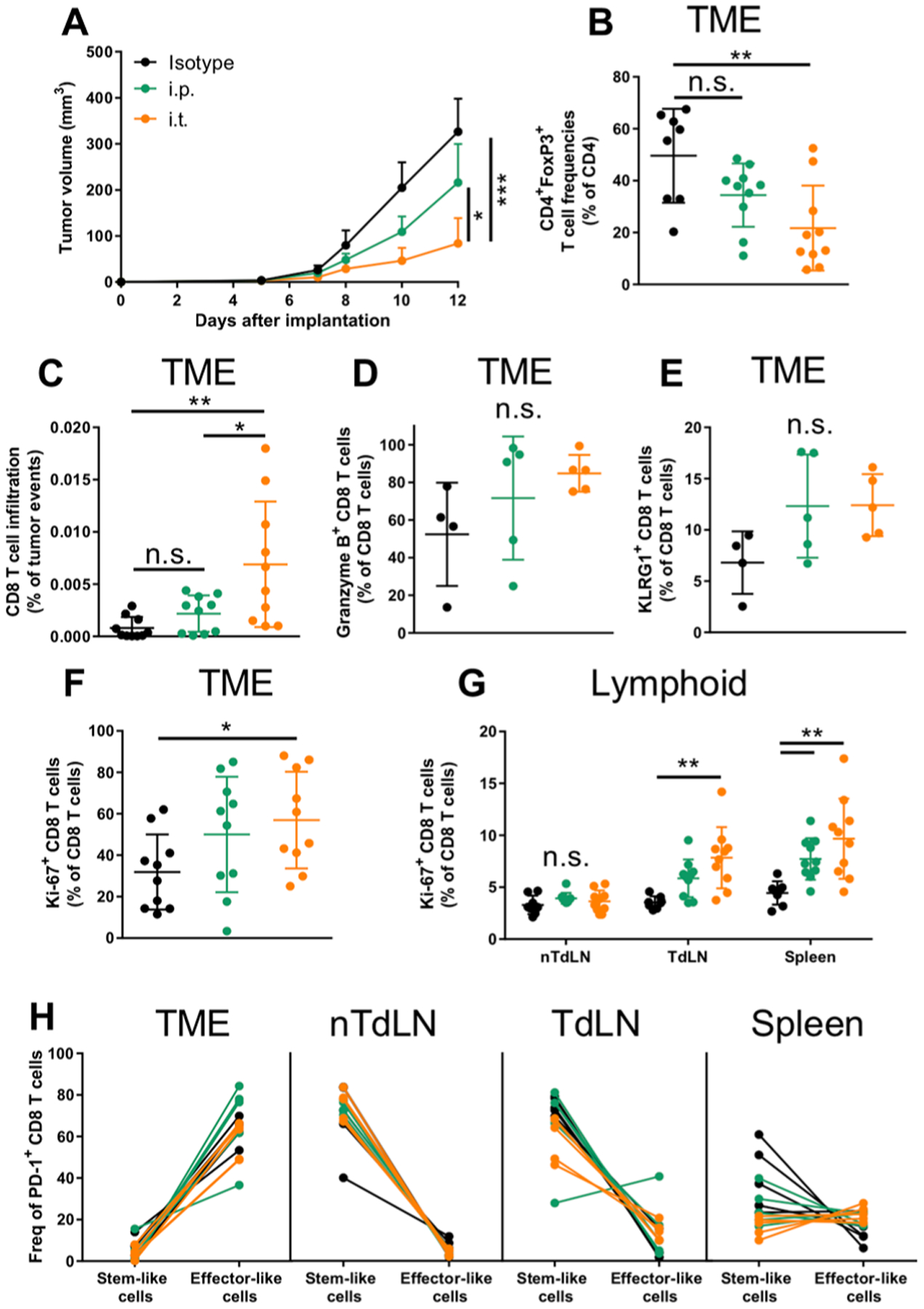
Tumor-bearing mice intratumorally administered ICB exhibit distinct T cell changes in TME, TdLN, and spleen. (**A**) B16F10 tumor growth over 12 days with 150 μg of each ICB mAb [combination of aPD-1 and aCTLA-4 (9H10)] administered on days 5, 7, and 9 after tumor implantation. (**B**) Frequencies of CD4^+^FoxP3^+^ T cells. (**C**) Frequencies of CD8^+^ T cells in TME. Frequencies of granzyme B^+^ (**D**) and KLRG1^+^ (**E**) CD8^+^ T cells in TME. Frequencies of Ki-67^+^CD8^+^ T cells in TME (**F**) and lymphoid tissues (**G**). (**H**) Frequencies of “stem-like” (Tcf1^+^Tim3^−^) versus “effector-like” (Tcf1^−^Tim3^+^) CD8 T cells, pregated on PD-1^+^ cells, in the TME, nTdLNs, TdLNs, and spleen. (B to H) End point analyses of tissues on day 12. (D) to (F) represent one independent experiment (*n* = 5); (A) to (C), (F), and (G) represent two independent experiments (total *n* = 10). Statistical analyses were done using ANOVA with Tukey’s test. *P < 0.05, **P < 0.01, and ***P < 0.001. n.s., not significant. Data are represented by means + SEM (A) or ± SD (B to G).

The immunological mechanisms underlying the therapeutic responses seen with i.t. and i.p. ICB therapy (aCTLA-4 and aPD-1 in combination) were explored. T cell phenotypes in i.t. saline (control)–or ICB-treated animals bearing single B16F10 tumors were analyzed 12 days after tumor implantation. Administration i.t. led to a reduction in tumor burden (Fig. 2A) and was associated with a reduction in trT_reg_ frequencies (Fig. 2B), which can be attributed to the particular aCTLA-4 mAb clone used (4–6). ICB therapy was also associated with an increase in CD8 TILs, although this effect was limited to i.t. administration here (Fig. 2C). Of these CD8^+^ TILs, the frequencies of granzyme B–producing (Fig. 2D) and effector [killer cell lectin-like receptor G1–positive (KLRG1^+^)] cell frequencies (Fig. 2E) were similar between i.p. and i.t. administration, suggesting that effective therapy is associated with increased frequencies of CD8 TILs rather than reinvigoration of exhausted TILs. Cycling CD8 T cell frequencies were found to be increased in the TME and TdLN using i.t. administration, with comparable increases observed in the spleen between i.p. and i.t. administration, and with no changes observed in the non-TdLNs (nTdLNs) (Fig. 2, F and G). Similar results for Ki-67^+^ frequencies were also observed in the CD4 T cell compartment (fig. S1). Frequencies of CD8 T cells within each tissue compartment exhibiting stem-like (PD-1^+^Tcf1^+^Tim3^−^) versus effector-like (PD-1^+^Tcf1^−^Tim3^+^) CD8 T cell phenotypes were also assessed. The phenotypes of activated (PD-1^+^) CD8 T cells were predominately effector-like in the TME compared to stem-like in the LNs, with a balance in-between in the spleen regardless of therapy or route of administration (Fig. 2H). Of note, ex vivo staining confirmed that therapeutic aPD-1 mAb did not block the binding of aPD-1 mAb used for flow cytometry staining (fig. S2). These data support the concept that ICB efficacy is, in part, mediated by increasing the frequencies of TILs that may originate from the TME or peripheral tissues including the TdLN or spleen.

### Administration route affects mAb biodistribution

**Fig. 3.**
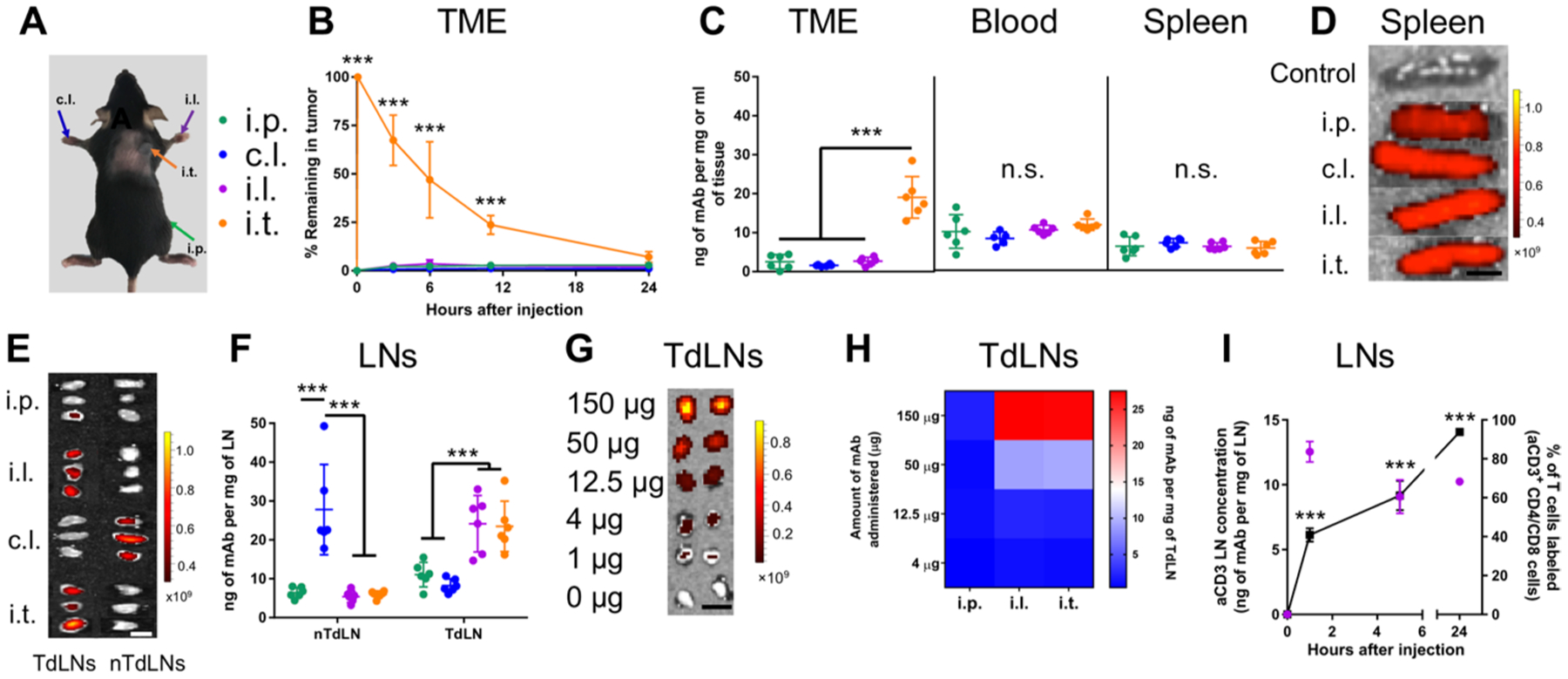
Directed mAb delivery to various tissue combinations with different routes of administration. Measured tissue concentrations of Alexa Fluor 647–labeled aPD-1 or aCTLA-4 (9H10) mAb. (**A**) Injection sites and color scheme. (**B**) mAb signal (IVIS quantification) in TME over 24 hours after injection. (**C**) mAb concentration in tumor, blood, and spleen 24 hours after injection. (**D** and **E**) Representative IVIS images of mAb accumulation in spleens (scale bar, 0.5 cm) (D) and LNs (scale bar, 0.25 cm) (E). (**F**) mAb concentrations in LNs 24 hours after injection. (**G** and **H**) Measured concentrations of Alexa Fluor 647–labeled aPD-1 or aCTLA-4 in TdLNs using different mAb doses. (G) Representative IVIS images of mAb accumulation in TdLNs after i.l. administration (scale bar, 0.25 cm). (H) Quantification of (G). (**I**) Concentration of aCD3 (purple, left axis) and frequencies of T cell labeling of injected aCD3 (black, right axis) in LNs draining forelimb i.d. injection. Data represent two independent experiments (total *n* = 5). Statistical analyses were done using ANOVA with Tukey’s test. ***P < 0.001; n.s., not significant. Data are represented by means ± SD.

Considering that responses were observed in secondary lymphoid tissues in addition to the TME after i.t. administration, we assessed the effect of route of administration on mAb accumulation within the spleen, LNs (tumor-draining or nondraining), and TME as well as other systemic tissues. Alexa Fluor 647–labeled aPD-1 or aCTLA-4 mAbs (fig. S3A) were measured after a single dose using four different administration routes: i.p., in the forelimb skin contralateral to the tumor (c.l.), in the forelimb skin ipsilateral to the tumor (i.l.), and i.t. 5 days after B16F10 tumor implantation (Fig. 3A). Tumor accumulation of mAb was sustained over 24 hours using an i.t. injection but was low to negligible using other administration routes (Fig. 3, B and C). mAb concentrations in the blood and spleen were also equivalent between administration routes (Fig. 3, C and D). When assessing mAb accumulation in LNs, i.p. administration resulted in minimal accumulation in any measured LN, whereas c.l. administration led to accumulation within nTdLNs (Fig. 3, E and F). Using i.l. and i.t. administration led to detectable concentrations of mAb solely within TdLNs (Fig. 3, E and F). These locoregional administration routes allowed for reduced dosing while maintaining mAb accumulation within TdLNs (Fig. 3, G and H). Accumulation of mAb in dLNs was not an effect of dye labeling, because administered nonfluorescent mAb accumulated within dLNs as with Alexa Fluor 647–tagged mAb (fig. S3B). Using these four different routes of administration allowed for subsequent studies to explore the effects on drugging particular tissues of interest and their effects on ICB therapeutic efficacy.

To explore whether lymph-delivered mAb had access to LN T cells, aCD3 (in place of immune checkpoint targeting) mAb was administered in the forelimb to target LN-resident CD3-expressing T cells. A gradual increase in T cell labeling of aCD3 mAb was observed over 24 hours, with nearly 100% of T cells labeled with Alexa Fluor 647–aCD3. Because comparable total LN mAb concentrations measured in LN tissue homogenates were observed within LNs at all time points (Fig. 3I) and this measured LN mAb concentration was sufficient to label ~100% of T cells when LNs were mechanically and enzymatically degraded after resection (fig. S3C), this suggests that labeling of T cells by i.d. administered aCD3 mAb within intact LNs is a diffusion-limited, intra-LN transport process. These results are in line with a recently published study, confirming that mAb has access to LN cells (38). Overall, these results demonstrate that various administration routes can be used to direct the delivery of mAb to T cells in specific tissues, including the TME, LNs, spleen, and blood.

### TdLN-targeted ICB improves anti-melanoma response

**Fig. 4.**
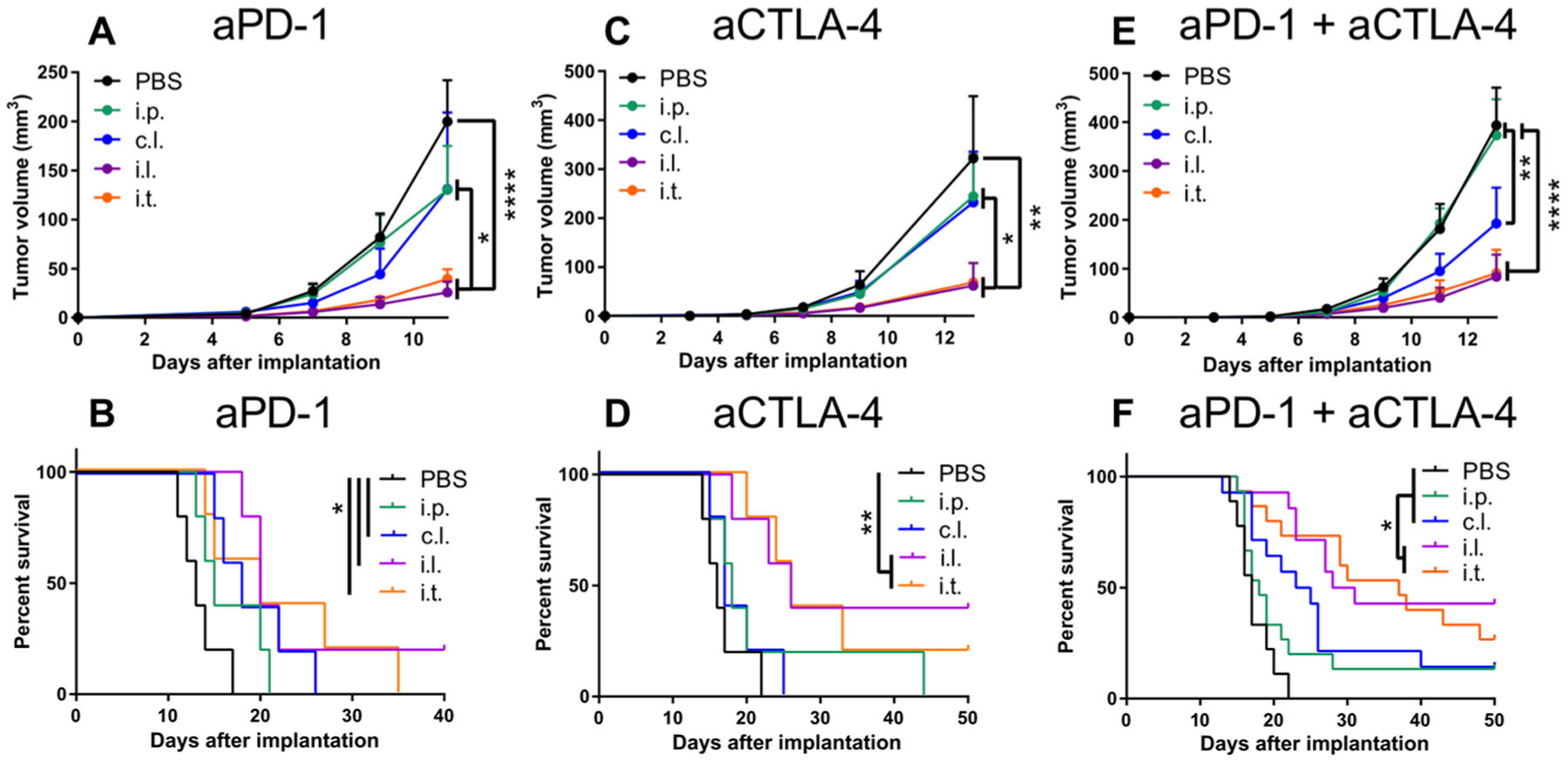
ICB directed toward TdLNs potentiates ICB therapeutic effects in melanoma. B16F10 tumor growth and animal survival after aPD-1 monotherapy (**A** and **B**), aCTLA-4 (9H10) monotherapy (**C** and **D**), and aPD-1 + aCTLA-4 (9H10) therapy (**E** and **F**) using 150 μg of each mentioned mAb. Tumor growth is shown in (A), (C), and (E), and animal survival is shown in (B), (D), and (F). (A) to (D) represent one independent experiment (*n* = 5); (E) and (F) represent three independent experiments (total *n* = 15). Statistical analyses were done using ANOVA with Tukey’s test. Log-rank (Mantel-Cox) test for survival curves. *P < 0.05, **P < 0.01, and ****P < 0.0001. Data are represented by means + SEM.

To elucidate the mechanistic effects of modulating immune checkpoints in various tissues on antitumor immunity, ICB mAbs were administered using the injection routes/locations depicted in Fig. 3A. This approach allowed the effects of ICB in specific tissues to be decoupled because i.t. administration results in appreciable mAb accumulation within the TME, TdLN, and spleen; i.d. forelimb injections target only the TdLN or nTdLN and spleen; and i.p. administration results in accumulation only in the spleen but not in the TME or in LNs. On days 5, 7, and 9 after B16F10 melanoma implantation, mice were treated with various ICB therapeutic regimens, including aPD-1, aCTLA-4, or the combination of the two. When used as monothera-pies, aPD-1 and aCTLA-4 mAb administered i.p. and in the forelimb c.l. to the tumor had no effect on tumor growth and animal survival, whereas administration of ICB in the forelimb i.l. to the tumor and i.t. reduced tumor growth during treatment to equivalent extents, which in the case of aCTLA-4 monotherapy led to prolonged survival (Fig. 4, A to D). However, ICB therapy was less effective in larger-sized tumors (fig. S4). Similar effects were observed when aPD-1 was combined with aCTLA-4 (Fig. 4, E and F). Overall, these data suggest that targeting of TdLNs (in addition to the spleen) results in the generation of robust antitumor immunity much greater than that seen with systemic administration.

### Locoregional ICB promotes tumor immunity in lymphoid tissues

**Fig. 5.**
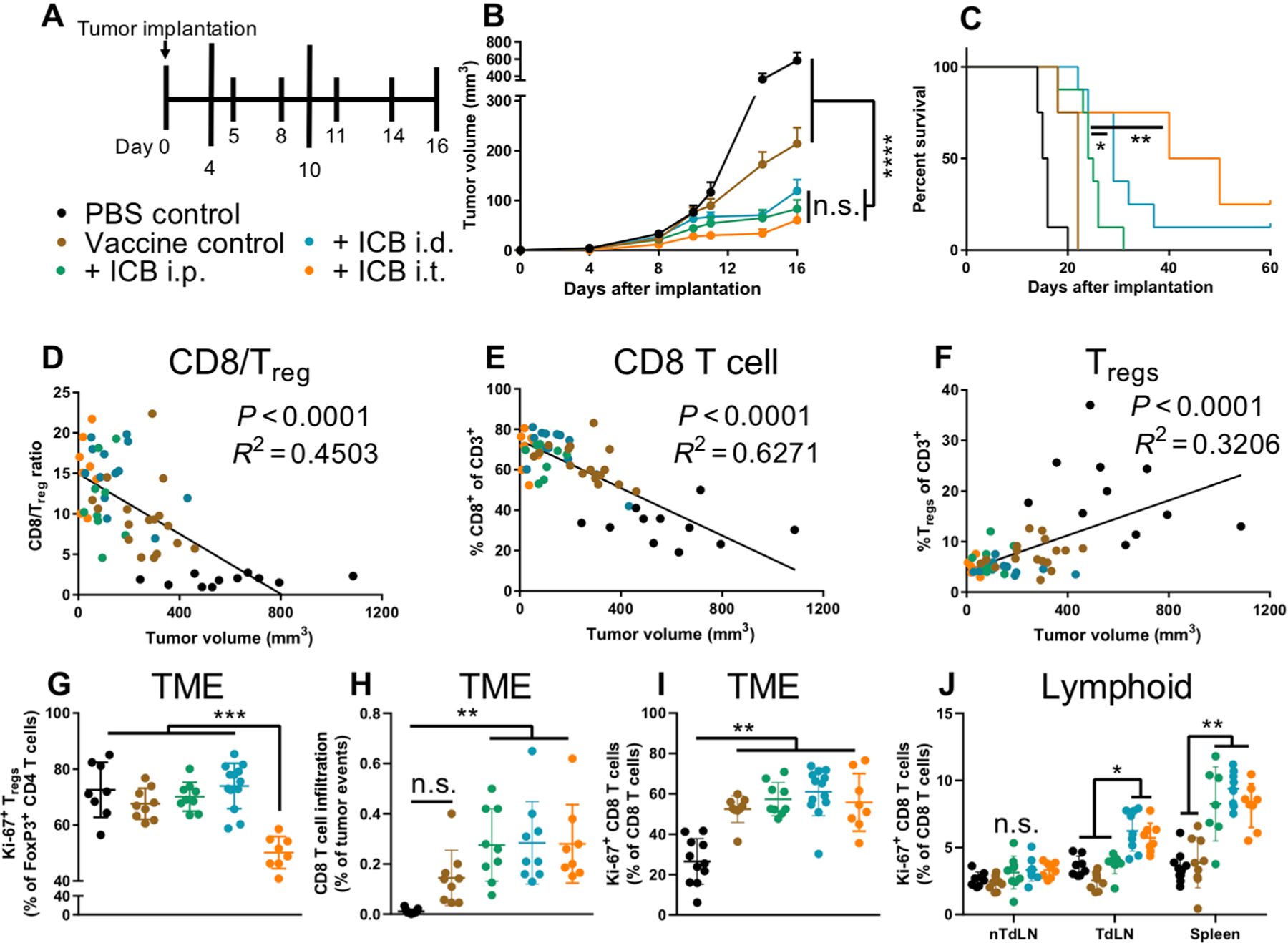
ICB directed to TdLNs alone or in combination with the TME improves therapeutic effects of vaccination. (**A**) B16F10-OVA treatment schedule and color scheme. Vaccination was performed by i.d. administration of 3 μg of CpG and 10 μg of OVA in each limb on days 4 and 10. One-hundred fifty micrograms of each ICB mAb [aPD-1 and aCTLA-4 (9H10) in combination] using the indicated administration routes on days 5, 8, 11, and 14. (**B**) Tumor growth during the treatment window. (**C**) Animal survival curves. (**D** to **F**) Tumor volume (*x* axis) versus T cell infiltration (*y* axis): (D) CD8^+^/CD4^+^FoxP3^+^ TIL ratio, (E) CD8^+^ frequency of CD3^+^ TILs, and (F) CD4^+^FoxP3^+^ frequency of CD3^+^ TILs. (**G**) Frequencies of Ki-67^+^CD4^+^FoxP3^+^ in TME. (**H**) Frequencies of CD8^+^ T cells in TME. Frequencies of Ki-67^+^CD8^+^ T cells in TME (**I**) and lymphoid tissues (**J**). (B) represents three independent experiments (total *n* = 14); (C) represents one (vaccine control) or two (all groups excluding vaccine control) independent experiments (total *n* = 4 to 8); (D) to (J) represent two (vaccine control, ICB i.p., and ICB i.t.) or three (PBS control and ICB i.d.) independent experiments (total *n* = 8 to 14). Statistical analyses were done using ANOVA with Tukey’s test. Log-rank (Mantel-Cox) test for survival curves. *P < 0.05, **P < 0.01, ***P < 0.001, and ****P < 0.0001. Data are represented by means + SEM (B) or ± SD (G to J).

We next explored the effects of ICB therapy in combination with a model tumor vaccine. The rationale was to develop and expand a robust antitumor CD8 T cell pool in tumor-bearing animals before modulation of T cell activation and effector functions resulting from ICB. Mice bearing B16F10 melanomas expressing ovalbumin (OVA) were vaccinated i.d. in each limb with OVA protein as tumor antigen and CpG oligodeoxynucleotide (CpG) as an adjuvant 4 and 10 days after tumor implantation and before ICB mAb administration (aPD-1 and aCTLA-4 in combination) on days 5, 8, 11, and 14 (Fig. 5A). Irrespective of the route of mAb administration, ICB improved vaccine effects during treatment, as measured by tumor outgrowth over the first 16 days (Fig. 5B). After cessation of therapy, ICB administered in the skin (either i.t. or i.d.) conferred improved survival (Fig. 5C) relative to that of systemically administered ICB (i.p.). T cell phenotyping on day 16 after tumor implantation revealed that increased CD8/T_reg_ ratios and CD8 T cell infiltration correlated with smaller tumors (Fig. 5, D and E), whereas increased T_reg_ frequencies correlated with increased tumor size (Fig. 5F). ICB administered i.t. reduced proliferating T_regs_ (CD4^+^FoxP3^+^Ki-67^+^) within the tumor compared to other ICB administration methods (Fig. 5G), an effect attributable to the particular aCTLA-4 mAb isotype used (4–6) and higher CTLA-4 surface expression on T_regs_ (fig. S5). When mice were treated with ICB therapy, increased infiltration of CD8 TILs was observed (Fig. 5H). However, similar rates of CD8 TIL proliferation were observed regardless of therapy or route of administration (Fig. 5I). Instead, increases in proliferation were observed in lymphoid tissues, specifically the TdLN using an i.d. or i.t. administration and the spleen with the addition of ICB therapy (Fig. 5J). Similar to results exploring the cell state of PD-1^+^ CD8 T cells (Fig. 2H), the PD-1^+^ CD8 T cells within the TME were predominately effector-like cells, whereas lymphoid tissues consisted of both effector- and stem-like CD8 T cells (fig. S6, A to D). Furthermore, CD8 T cells generated in LNs and spleen with ICB therapy were functional and capable of responding to tumor antigen upon ex vivo restimulation (fig. S6, E and F). Similar patterns were observed in the CD4 helper compartment (fig. S7). Together, these results are in line with neoadjuvant studies demonstrating that improved responses are associated with increased CD8 T cell proliferation and tumor infiltration (Fig. 2), which were achieved via concurrent drug modulation of immune checkpoint pathways in the TME and LNs.

### TdLN- and TME-directed ICB enable dose sparing

**Fig. 6.**
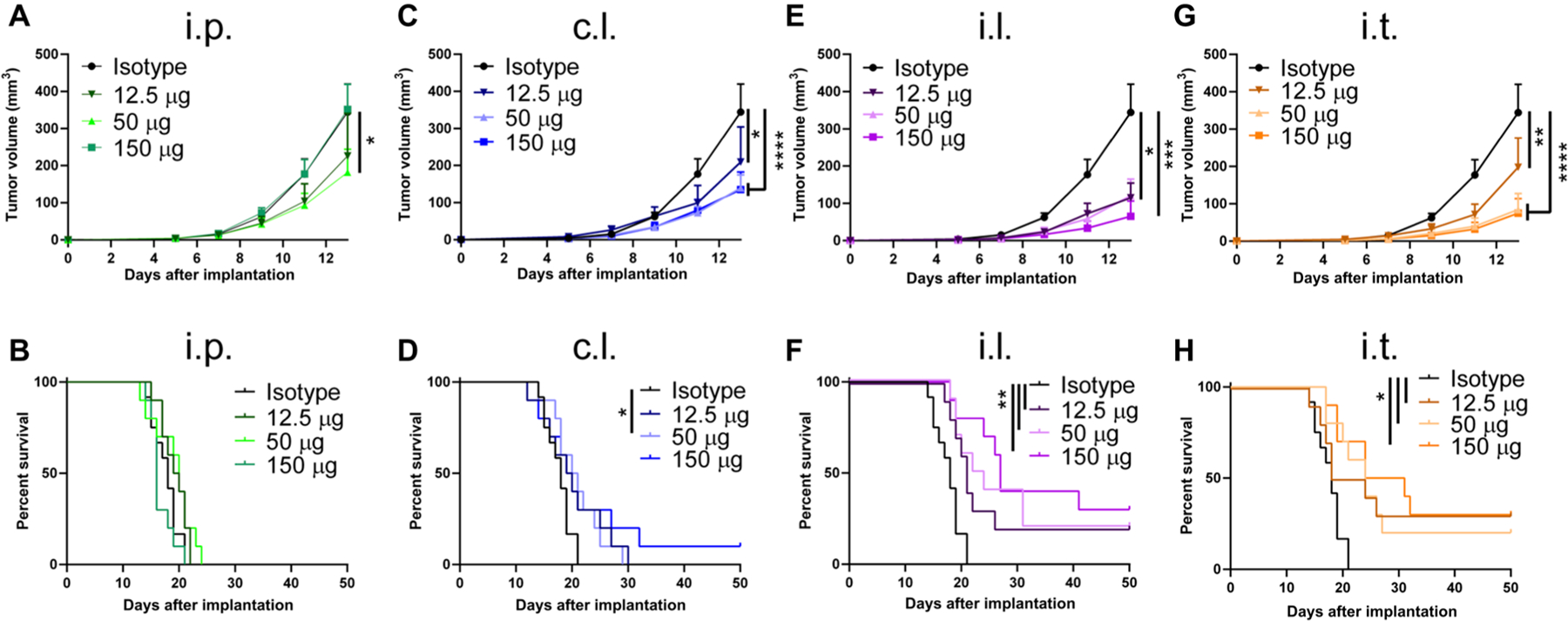
ICB directed to TdLNs alone or in combination with the TME potentiates ICB therapeutic effects independent of trT_reg_ depletion. B16F10 tumor growth and animal survival after ICB therapy using 150, 50, or 12.5 μg of each aPD-1 (clone RMP1–14) in combination with aCTLA-4 (clone 4F10); (**A** and **B**) i.p. administration, (**C** and **D**) c.l. administration, (**E** and **F**) i.l. administration, and (**G** and **H**) i.t. administration. Tumor growth is shown in (A), (C), (E), and (G), and animal survival is shown in (B), (D), (F), and (H). Combined data of two independent repeats (total *n* = 10). Statistical analyses were done using ANOVA with Tukey’s test. Log-rank (Mantel-Cox) test for survival curves. *P < 0.05, **P < 0.01, ***P < 0.001, and ****P < 0.0001. Data are represented by means +SEM.

Considering the dose-toxicity relationship of ICB therapy, we assessed ICB efficacy in a dose de-escalation study using aPD-1 and aCTLA-4 (fig. S8). Dose-dependent effects were observed in the case of nTdLN (c.l.) and TdLN (i.l.) delivery, whereas i.t. administration did not display a dose-efficacy relationship (fig. S8). This may be explained by our observed reductions in trT_reg_ after following i.t. administration (Figs. 2B and 5G), which is in line with multiple other reports showing that the efficacy of aCTLA-4 therapy is due, in part, to the depletion of trT_regs_ when an IgG2a clone is used (4–6). However, T_reg_ depletion by aCTLA-4 has not been observed in the clinical setting, and instead, mAb clones of aCTLA-4 used in human patients have been shown to act predominately via CTLA-4 receptor blockade and favoring CD28 ligation (39, 40). We therefore investigated the therapeutic effects of CTLA-4 blockade using a mAb clone of an IgG1 isotype (4F10) using an identical dosing schedule to those conducted using the T_reg_-depleting (IgG2a isotype) aCTLA-4 mAb clone (Fig. 4). Using this non–T_reg_-depleting aCTLA-4 mAb clone (IgG1), i.p. and c.l. administration had small antitumor therapeutic effects (Fig. 6, A to D). Conversely, both i.l. and i.t. administration elicited robust anti-tumor therapeutic effects even at the lowest tested dose (12.5 μg; Fig. 6, E to H). These data thus demonstrate that the benefits of LN targeting are applicable to multiple aCTLA-4 mAb clones with differing immunomodulatory mechanisms. These results suggest that the efficacy of ICB directed to the TdLNs alone or in addition to the TME is roughly equivalent, at least at the doses tested in this model, pointing to LNs mediating the expansion of CD8 T cell immunity in response to ICB.

### Directing ICB to TdLNs improves therapeutic effects in breast cancer

**Fig. 7.**
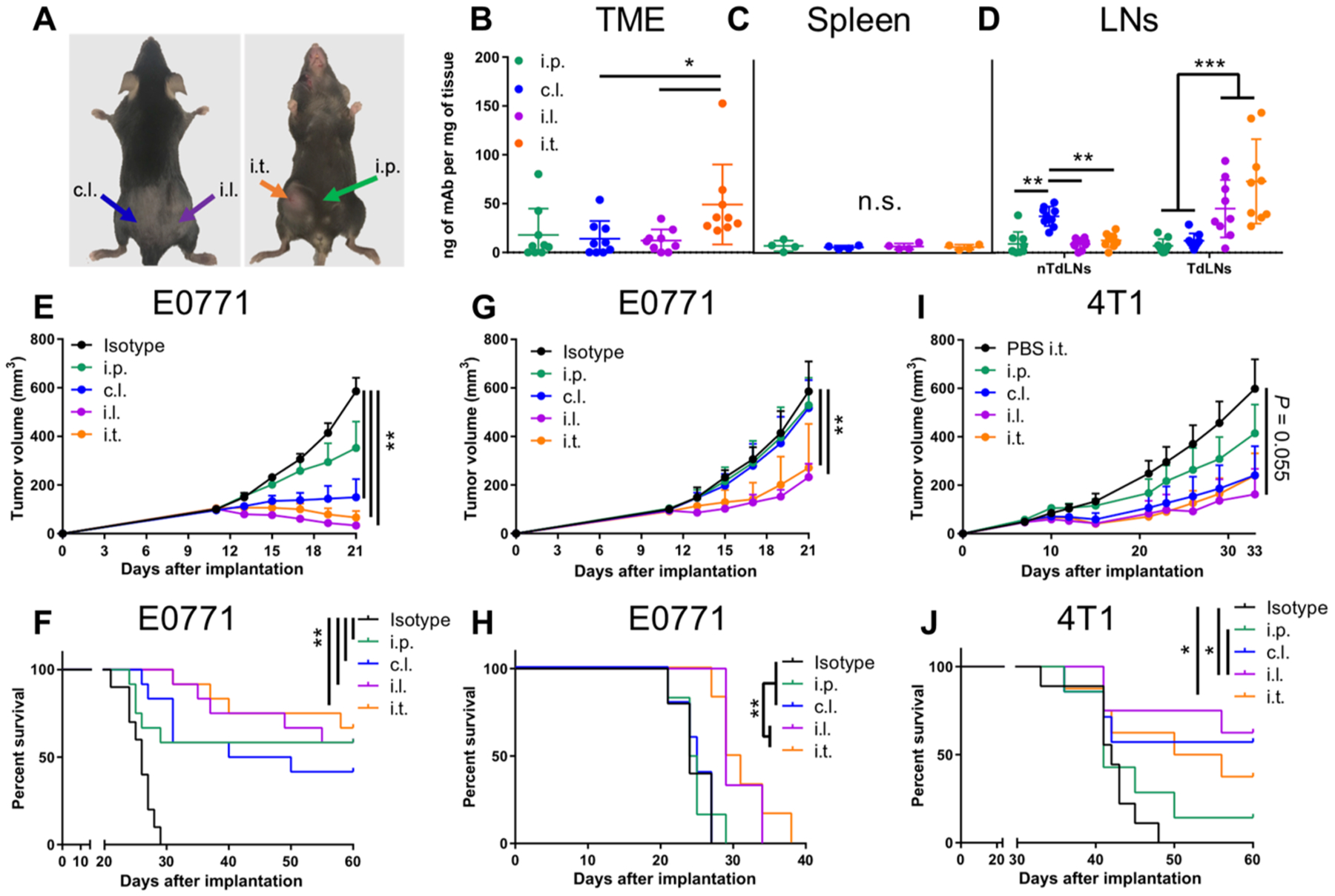
ICB directed to TdLNs elicits robust antitumor therapeutic effects in breast tumor models. (**A**) Image of administration sites and color scheme. mAb concentrations in (**B**) TME, (**C**) spleen, and (**D**) LNs in E0771 tumor-bearing animals 24 hours after injection. (**E**) Growth curves of E0771 tumors treated with a single 100-μg dose of each aPD-1 and aCTLA-4 (clone 9H10) when tumors reached approximately 100 mm^3^. (**F**) Survival of animals treated in (E). (**G**) Growth curves of E0771 tumors treated with 30 μg of each aPD-1 and aCTLA-4 (clone 4F10) therapy on days 10, 14, and 20. (**H**) Survival of animals treated in (G). (**I**) Growth curves of 4T1 tumors treated with 50 μg of each aPD-1 and aCTLA-4 (clone 4F10) on day 7. (**J**) Survival of animals treated in (I). (B) and (D) to (F) represent two independent experiments (total *n* = 9 to 11); (C) and (G) to (J) represent one experiment (total *n* = 4 to 8). Statistical analyses were done using ANOVA with Tukey’s test. Log-rank (Mantel-Cox) test for survival curves. *P < 0.05, **P < 0.01, and ***P < 0.001; n.s., not significant. Data are represented by means + SEM (E, G, and I) or ± SD (B to D).

To extend these results beyond melanoma models, two different mammary carcinoma models, E0771 and 4T1, were implanted orthotopically in the fourth mammary fat pad. These implantation sites generate TdLNs differing from those generated using the dorsal lateral B16F10 melanoma model, specifically the ipsilateral inguinal (primary draining) and axillary (secondary draining) LNs. To deliver mAb to TdLNs or nTdLNs, mAb administration was performed in the flank skin of mice (Fig. 7A), whereas the systemic i.p. administration was kept the same and i.t. administration consisted of an injection into the mammary fat pad tumor site. After administration of fluorescently labeled mAb (aPD-1), i.t. administration resulted in sustained mAb retention in the TME, whereas other administration routes resulted in low to minimal TME concentrations (Fig. 7B). Accumulation of mAb in the spleen was equivalent regardless of administration route (Fig. 7C). Contrastingly, i.l. and i.t administration of mAb (aPD-1 or isotype) led to higher TdLN accumulation, whereas c.l. administration led to accumulation within nTdLN (Fig. 7D).

Using aPD-1 in the E0771 model, locoregional therapy was as effective as systemic (i.p.) administration (fig. S9, A and B), motivating the exploration of aPD-1 in combination with aCTLA-4. To this end, a single dose of aPD-1 in combination with aCTLA-4 (clone 9H10) administered i.t., i.l., and c.l. resulted in reductions in tumor growth compared to either no treatment or systemic i.p. administration (Fig. 7E). Improvements in survival were found for this combination therapy (40 to 60% overall complete response) and were comparable between all administration routes (Fig. 7F). Dose de-escalation studies demonstrated that survival was dose sensitive, but effects were again roughly equivalent between ICB mAb administration route (fig. S9, C to F). These data demonstrate that the therapeutic benefits of ICB with aPD-1 in this model can be improved with aCTLA-4, which is expected given this aCTLA-4 mAb clone’s pleotropic effects on the antitumor immune response.

To decouple the effects of trT_reg_ depletion and expansion of CD8 T cell immunity associated with aCTLA-4 mAb treatment, we evaluated the effects of the 4F10 mAb clone of aCTLA-4 that does not result in trT_reg_ depletion (Fig. 7, G and H). Antitumor therapeutic efficacy of all tested administration routes overall was much less effective, indicative of a major role that T_regs_ play in the immune physiology of the E0771 model. However, i.l. and i.t. administration did suppress tumor growth and prolong survival compared to i.p. and c.l. administration (Fig. 7, G and H). Effects of aPD-1 mAb in combination with aCTLA-4 mAb (clone 4F10) in the highly metastatic 4T1 model were also tested. Improved responses were observed with c.l., i.l., and i.t. therapy compared to that of systemic i.p. therapy (Fig. 7, I and J), with no signs of metastasis in responding mice (fig. S9, G and H). When the trT_reg_-depleting aCTLA-4 clone (clone 9H10) was used, systemic therapy was just as effective as administration in the skin (both i.l. and c.l.; fig. S9, I and J). These data suggest that these breast tumor models are highly infiltrated with T_regs_ implicated in tumor progression. Nevertheless, as in the B16F10 melanoma model, (Td)LN targeting of mAb improved therapeutic responses to ICB in two breast tumor models compared to conventional systemic administration.

### Locoregional administration reduces toxicities of ICB

**Fig. 8.**
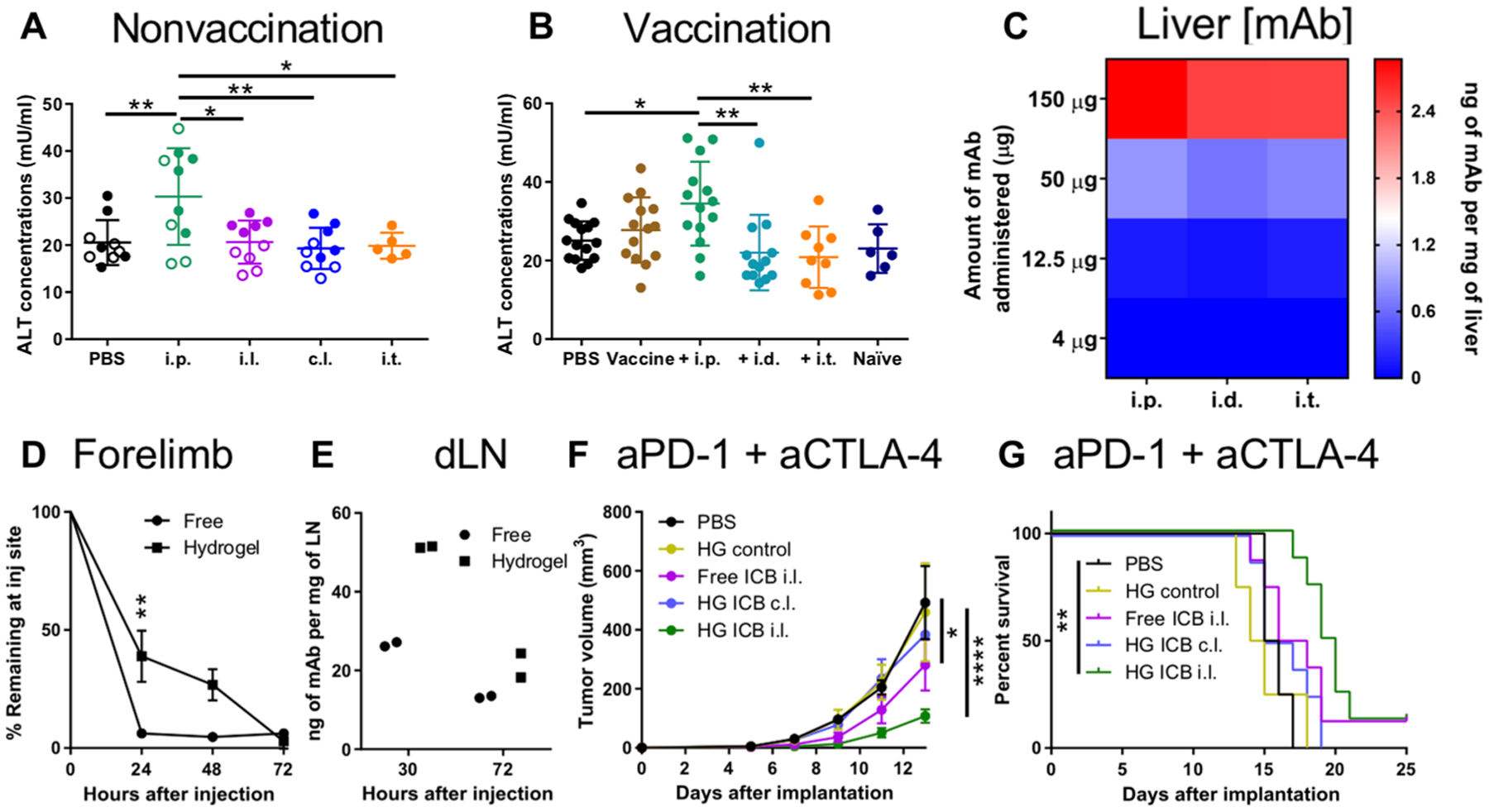
Locoregional administration reduces ICB-associated toxicities and improves TdLN-dependent effects of sustained mAb release. Serum alanine transaminase (ALT) concentrations 12 days after B16F10 implantation and 22 days after E0771 implantation (**A**) and 16 days after tumor implantation in vaccinated B16F10-OVA–bearing animals (**B**). (A) Closed circles, B16F10; open circles, E0771. Naïve: tumor-free mouse. PBS was administered i.t. (**C**) Liver mAb concentrations 24 hours after i.p. administration at various total doses. (**D**) mAb signal (IVIS quantification) in forelimb over 72 hours after injection. (**E**) dLN mAb concentrations 30 and 72 hours after injection. B16F10 tumor growth (**F**) and animal survival (**G)** after ICB therapy with 25 μg of each aPD-1 (clone RMP1–14) and aCTLA-4 (clone 9H10). (A) represents one experiment in each tumor model (total *n* = 10); (B) represents two (PBS control, vaccine control, ICB i.p., and ICB i.d.) or three (ICB i.t. and naïve) independent experiments (total *n* = 6 to 16); (C) to (G) represent one experiment [C and E, *n* = 2; D, *n* = 4; F and G, *n* = 4 (controls) or *n* = 8 (ICB groups)]. Statistical analyses were done using ANOVA with Tukey’s test. *P < 0.05, **P < 0.01, ***P < 0.001. Data are represented by means + SEM (F) or ± SD (A, B, and D).

ICB is associated with immune-related adverse events (iRAEs) that can lead to discontinuation of treatment, especially when aPD-1 and aCTLA-4 mAb therapies are used in combination (41). To explore ICB-related toxicities, blood was collected 2 to 3 days after the last mAb administration, and the serum was analyzed. When used with ICB alone, i.p. administration led to increased alanine transaminase (ALT) serum concentrations compared to no treatment or cutaneous injections in both the B16F10 and E0771 tumor models (Fig. 8A). Similar effects of locoregional therapy on ALT serum concentrations were also observed when mice were vaccinated (Fig. 8B). Furthermore, mAb concentrations in the liver, kidneys, and lungs were proportional to the administered dose of ICB mAb (Fig. 8C and fig. S10). Overall, these results suggest that locoregional administration of ICB mAb, which can elicit robust immunity and antitumor efficacy, reduces the toxicity associated with systemic and high-dose ICB therapy.

### Controlled-release mAb formulation drugging TdLN improves effects of locoregional ICB

We explored the effects of sustained LN drugging through the use of a hydrogel formulation to improve local pharmacokinetics and reduce the need for multiple injections. Formed from biocompatible, U.S. Food and Drug Administration (FDA)–approved block co-polymer Pluronic F-127, equivalent hydrogel formulations have been used to prolong injection site protein retention (42) to improve drug bioactivity. After injection of fluorescently labeled mAb, the hydrogel formulation prolonged mAb retention at the injection site to over 72 hours, leading to higher mAb concentrations in the dLN after injection compared to those resulting from administration of free (no polymer) mAb solution (Fig. 8, D and E). After a single injection of both aPD-1 and aCTLA-4 mAb on day 5 after tumor implantation in the B16F10 model, the hydrogel formulation injected in the forelimb i.l. to the tumor afforded improved antitumor efficacy, effects not seen when it was injected in the c.l. limb (Fig. 8, F and G). Furthermore, the hydrogel formulation improved efficacy relative to that of the free, unformulated mAb, suggesting that sustained mAb accumulation within TdLNs improves effects (Fig. 8, F and G). Overall, these results demonstrate the potential for controlled-release strategies directing mAb delivery into the lymphatic-draining tissue basin co-draining the tumor to improve the therapeutic effects of ICB.

## DISCUSSION

ICB has emerged as a promising class of anticancer therapy, but these treatments are associated with low response rates and substantial toxicities, which may be related to systemic administration of these drugs. Targeting the TME versus LNs and spleen using different administration routes/doses/formulations was explored to increase mAb accumulation within these tissues and therefore modulate these pathways at the effector and priming phases, respectively. In three tumor models, varying from poorly to highly responsive to ICB, administration routes that mediate mAb accumulation in (Td)LNs led to superior therapeutic effects on tumor control compared to those achieved by systemic administration.

An abscopal effect was observed with tumor-localized ICB using i.t. administration, demonstrating the generation of an antitumor immune response that is systemically functional. This is suggestive of tumor-localized therapy being capable of expanding endogenous antitumor immunity, given observations of higher TIL frequencies. Studies have indicated that increasing frequencies of CD8 TILs improve response to immunotherapy and patient survival (14, 18). Whether they originate from the TME or elsewhere before migrating into the TME remains unclear. However, tumor-specific T cells have been found in the blood after ICB treatment, suggesting the latter (17, 43). Here, we observed increased frequencies of proliferating CD8 T cells not only in the TME but also in the TdLN and spleen after i.t. treatment, suggesting that TILs may originate from multiple tissue sites. We did observe stem-like CD8 TILs; however, the predominant phenotype of activated CD8 TILs was effector-like, which may be due to proliferation and differentiation of tumor-resident stem-like CD8 T cells (44). In line with this, stem-like CD8 TILs reside in APC-enriched niches that support their function, and loss or absence of these niches is associated with disease progression (45). In addition to the TME, we observed stem-like CD8 T cells in secondary lymphoid tissues, consistent with previous reports (11, 44), including human LNs (26). Thus, secondary lymphoid tissues are a potential source of tumor-killing effector-like CD8 T cells. Accumulation of mAb within the spleen and LNs was also associated with expansion of the effector-like cell pool within these tissues. Poorly immunogenic TMEs lacking APC niches or TILs may therefore not respond to systemic ICB therapy, and may instead benefit from targeted delivery of ICB mAbs into lymphoid tissues where these stem-like CD8 T cells reside at high frequencies. Overall, our results support the conclusions that ICB therapy increases TIL frequencies and that, because TILs may originate outside the TME, lymphoid tissues represent potential tissue targets for ICB modulation.

The effect of LN-directed mAb delivery was found to be beneficial in multiple therapeutic settings. In the B16F10 melanoma model, systemic i.p. administration led to minimal therapeutic efficacy that may be due to poor delivery and accumulation of ICB mAbs in the TME and TdLNs. ICB therapy directed toward TdLNs via i.l. forelimb administration greatly improved response rates regardless of aCTLA-4 mAb clone used, which we hypothesize is due to improved T cell activation and subsequent infiltration into the TME. Dose de-escalation experiments revealed that ICB mAb directed to the TdLNs alone versus in combination with the TME via i.l. forelimb or i.t. administration, respectively, results in similar antitumor therapeutic effects. This is suggestive of the therapeutic benefits of ICB being conferred, at least partially, by activity within LNs, presumably at the APC:T cell synapse during the T cell priming phase. It may also be explained by the immune exclusion and poor immunogenicity of the B16F10 model. Put another way, drugging the TME does not appear to afford therapeutic effects when antitumor TILs are locally absent. This concept is in line with previous observations in melanoma models where knockout of T cell PD-1 expression does not improve tumor responses (12). This is further supported by our vaccination studies in the B16F10-OVA model, where ICB injected i.t. in combination with a tumor vaccine resulted in longer survival. Vaccination alone resulted in marked expansion and infiltration of antitumor T cells, thus providing local TILs for potential ICB modulation. Therefore, addition of ICB directly into the tumor, along with a reduction of proliferating trT_regs_, resulted in improved survival of i.t. treated mice. In line with neoadjuvant phenotyping results, addition of ICB therapy increased frequencies of TILs, which may be due to the higher frequencies of proliferating T cells in lymphoid tissues. This suggests that modulation of ICB in the spleen or TdLN may promote and sustain lymphocyte infiltration into the TME.

In breast tumor models, targeting mAb to (Td)LNs alone or in combination with the TME improved therapeutic benefits compared to systemic therapy. Notable differences were observed in the E0771 model when using a trT_reg_-depleting mAb clone of aCTLA-4 (9H10) versus a nondepleting clone (4F10), suggesting that these tumors are highly infiltrated with suppressive T_regs_ and/or that T_regs_ play a dominant role in immune-regulated disease progression in these models. An LN-directed drugging approach, which appears effective in eliciting robust T cell immunity, may thus need to be combined with other therapies to modulate such suppressive cell types to successfully combat breast cancer. Another consideration is that tumor physiology can vary greatly between tumor types and consequently affect mAb transport (34). Breast cancer models may have better mAb access to the TME from the blood relative to melanoma models, which may explain the i.p. efficacy observed. In the 4T1 model, c.l. administration improved treatment efficacy compared to systemic i.p. administration. This could be explained by the metastatic propensity and subsequent presence of tumor-associated antigen in tissues beyond the TME and TdLN including nTdLNs, thereby explaining the beneficial effects of nTdLN targeting.

When toxicity was explored, systemic i.p. administration increased the serum concentrations of ALT, whereas locoregional delivery did not. These data may be explained by a slower, more sustained delivery of ICB mAb into the circulation after cutaneous injection by virtue of clearance being mediated by lymphatic transport compared to a bolus delivery into the systemic circulation (46). Accumulation of mAb in systemic tissues was proportional to administered dose. This indicates that administration routes that afford dose sparing, such as injection into locoregional tissues, have the potential to minimize off-target toxicities.

There is interest in locoregional delivery of mAbs because systemic administration has several disadvantages, including cost and adherence (47). Local immune therapy via i.t. administration using aCTLA-4 and s.c. administration using aPD-1 has been reported for a variety of cancers including melanoma (36, 48). Here, we show that locoregional administration routes allow for efficient ICB mAb drugging of TdLNs to enable reduced dosing. This has advantages, including dose sparing to mitigate treatment toxicities and potential challenges associated with concentrating mAb solutions to accommodate the reduced injection volume relative to systemic infusions (~^1^/_10_), which can lead to protein aggregation, and therefore compromised efficacy, increased immunogenicity, and concerns for pharmacokinetic profiles (46). Moreover, this enables innovations in sustained mAb release strategies to be applied to ICB to reduce reliance on multiple injections and improve patient adherence. Because i.t. injections are not always feasible due to tumor size and internal location (48), locoregional administration targeting the LNs and not TME directly may also be advantageous as TIL frequencies are often low and exhausted T cells undergo epigenetic reprogramming that can limit TIL rejuvenation potential (49, 50). In addition, as noted above, tumor physiology is highly variable, which can negatively influence i.t. mAb diffusion and lymphatic transport (51). However, TdLNs may be challenging to identify and, in some cases, absent due to removal during LN biopsy/dissection, which may limit this approach to certain indications or neoadjuvant settings. Nevertheless, locoregional injection at a distant site from the tumor that drains to the same TdLNs may be of interest as an alternative to i.t. administration to broaden the number of patients who might benefit from a locoregional treatment approach and reduce treatment invasiveness.

Limitations of this work include T cell phenotyping not being restricted to known antigen-specific T cell clones. Toxicity at the nontumor injection site, which may promote activation of non–tumor-specific T cells and thereby contribute to iRAEs, also remains unexplored. Despite overall improved efficacy with TdLN-directed ICB therapy, responses remained variable, suggesting the need for additional or combination therapy approaches to improve overall rates of response. Benefits to ICB therapeutic efficacy conferred by locoregional administration may also be limited to disease contexts with smaller tumor burdens, which may limit translation into the clinic.

In conclusion, directing ICB mAbs to (Td)LNs by locoregional administration enhanced antitumor efficacy compared to systemically administered mAb and reduced associated toxicities in both melanoma and breast cancers. This simple approach requires no chemical modifications to the ICB mAbs, only reformulation, and may hold potential for clinical translation due to the current FDA approval, interest in patient compliance, and need to improve safety and response rates.

## MATERIALS AND METHODS

### Study design

This study was designed to explore the effects of targeting ICB mAb to LNs on antitumor efficacy in mouse melanoma and breast cancer models. We evaluated in what tissues immune checkpoint pathways were active and explored how ICB delivery to LNs differed from systemic therapy with ICB alone or in combination with vaccination. Sample sizes were chosen on the basis of previously published studies. For animal studies, mice were randomized into various groups before treatment, with each cage having one mouse per group. Experiments were not performed in a blinded fashion.

### Mice and cell lines

Cell lines were maintained in Dulbecco’s modified Eagle’s medium supplemented with 10% fetal bovine serum and 1% penicillin/streptomycin/amphotericin B and periodically checked for mycoplasma contamination. C57Bl6 and BalbC mice were purchased from The Jackson Laboratory. All protocols were approved by the Institutional Animal Care and Use Committee. Tumors were implanted intradermally in 6- to 12-week-old mice and monitored in anesthetized mice by caliper measurements of tumor width, length, and depth. Mice were euthanized when tumors ulcerated or reached 1.5 cm in any dimension.

### Treatment of B16F10 melanoma–bearing mice

The dorsal skin of C57Bl6 mice was shaved, and B16F10 or B16F10-OVA cells (10^5^) were implanted in the right dorsal flank on day 0. After 5 (when all tumors were visible), 7, and 9 days, mice were i.d. injected with 150, 50, or 12.5 μg of anti-mouse CTLA-4 (clone 9H10 or UC10–4F10–11; BioXCell) and/or rat anti-mouse PD-1 (clone RMP1–14; BioXCell) i.t., i.d. in the forelimb, or i.p. in 30 μl of saline. In abscopal tumor immunotherapy experiments, 10^5^ B16F10 cells were injected i.d. on the right dorsal skin of the mouse on day 0 and on the left dorsal skin on day 2. On days 5, 7, and 9, mice were injected with 150 μg of aCTLA-4 (clone 9H10) and aPD-1 (i.d. or i.p.) in saline. For immune cell phenotyping, mice were euthanized on day 12 and tissues were harvested. In vaccination studies, at 4 and 10 days, CpG (3 μg) and OVA (10 μg) were i.d. administered in 30 μl of saline in each limb. On days 5, 8, 11, and 14, mice received 150 μg of aCTLA-4 and aPD-1 mAb in 30 μl of saline either i.t., i.d. in the forelimb, or i.p. In studies evaluating the effects of sustained mAb release, Pluronic F127 (Sigma-Aldrich) was dissolved at 25 weight % in cold phosphate-buffered saline (PBS). Before injection, 25 μg of aCTLA-4 (clone 9H10) and aPD-1 mAb (5 μl) was mixed with 25 μl of the gel solution or PBS and i.d. injected once into one forelimb on day 5.

### In vivo mAb biodistribution studies

On day 5 after tumor implantation, mice were administered aCTLA-4 or aPD-1 mAb. Fluorescent imaging was performed with an IVIS Spectrum instrument (PerkinElmer) at the injection site over 24 hours. Twenty-four hours after mAb injection, mice were euthanized and tissues were collected for imaging and homogenization. Concentrations of mAb in homogenized tissues were determined using a standard curve of injected mAb solution in naïve tissue homogenates. Tissue background was subtracted from all measurements. For biodistribution experiments using aCD3 mAb, naïve mice were injected with 6.25 μg of mAb and euthanized after 1, 5, or 24 hours. LNs were either collagenase-treated for 30 min followed by tissue disruption to form single-cell suspensions (a process described in Supplementary Materials and Methods) or immediately cut up and dispersed into a single-cell suspension to prevent ex vivo T cell labeling.

### Treatment of E0771 and 4T1 breast cancer–bearing mice

E0771 (5 × 10^5^) or 4T1 (3.5 × 10^5^) cells resuspended in 30 μl of saline were implanted i.d. in the left mammary fat pad (fourth) in C57Bl6 or BalbC mice, respectively. For E0771 experiments, aPD-1 or aPD-1 and aCTLA-4 (clone 9H10) in combination were administered i.d. when tumors were ~100 mm^3^. Alternatively, 30 μg each of aPD-1 and aCTLA-4 (clone 4F10) mAb were administered on days 10, 14, and 17. For 4T1 experiments, 50 μg each of aPD-1 and aCTLA-4 (clone 4F10) mAb were administered on day 7 or 50 μg each of aPD-1 and aCTLA-4 (clone 9H10) mAb were administered on days 7 and 10. At end point, LNs and the spleen were harvested and imaged and organ sizes were measured using ImageJ.

### Statistics

Statistical significance of differences between experimental groups was calculated with Prism software (GraphPad). All data are expressed as means ± SD except for tumor growth (SEM). ****P < 0.0001, ***P < 0.001, **P < 0.01, and *P < 0.05 by unpaired two-tailed *t* tests or one- or two-way analysis of variance (ANOVA) followed by Tukey post hoc test for multiple comparisons. For survival curves, log-rank (Mantel-Cox) test was performed. Original data are provided in data file S1.

## Supplementary Material

Supplementary Material Materials and Methods
Fig. S1. Changes in CD4 T cell compartment resulting from ICB.
Fig. S2. Therapeutic and staining aPD-1 mAbs simultaneously stain PD-1-expressing T cells.
Fig. S3. mAb fluorescent labeling, accumulation within dLNs, and binding to LN-resident T cells after administration.
Fig. S4. ICB therapy is less effective in larger tumors.
Fig. S5. T_regs_ in TME express CTLA-4 at higher frequencies than helper CD4 and CD8 T cells.
Fig. S6. ICB therapy modulates CD8 T cells in various tissues leading to effector cell phenotypes.
Fig. S7. ICB with vaccination promotes CD4h activation in TdLNs.
Fig. S8. ICB directed to TdLNs alone or in combination with the TME enables dose sparing.
Fig. S9. Effective ICB therapy in breast cancers requires aCTLA-4.
Fig. S10. mAb accumulation in kidneys and lungs proportional to administered dose.
Data file S1. Individual data points for merged or averaged data plots.

## Data and materials availability:

All data associated with this study are present in the paper or the Supplementary Materials.
