# Supplementary material for: Blockade of immune checkpoints in lymph nodes through locoregional delivery augments cancer immunotherapy
Source: Sci Transl Med. Author manuscript; Available in PMC 2021 Sep 30. (PMC8377700; doi:10.1126/scitranslmed.aay3575)
Supplement: Supplementary Material — Materials and Methods Fig. S1. Changes in CD4 T cell compartment resulting from ICB. Fig. S2. Therapeutic and staining aPD-1 mAbs simultaneously stain PD-1-expressing T cells. Fig. S3. mAb fluorescent labeling, accumulation within dLNs, and binding to LN-resident T cells after administration. Fig. S4. ICB therapy is less effective in larger tumors. Fig. S5. Tregs in TME express CTLA-4 at higher frequencies than helper CD4 and CD8 T cells. Fig. S6. ICB therapy modulates CD8 T cells in various tissues leading to effector cell phenotypes. Fig. S7. ICB with vaccination promotes CD4h activation in TdLNs. Fig. S8. ICB directed to TdLNs alone or in combination with the TME enables dose sparing. Fig. S9. Effective ICB therapy in breast cancers requires aCTLA-4. Fig. S10. mAb accumulation in kidneys and lungs proportional to administered dose. Data file S1. Individual data points for merged or averaged data plots. [file NIHMS1730652-supplement-Supplementary_Material.pdf]

Science Translational Medicine
AAAS

stm.sciencemag.org/cgi/content/full/12/563/eaay3575/DC1

Supplementary Materials for

Blockade of immune checkpoints in lymph nodes through locoregional delivery augments cancer immunotherapy

David M. Francis, Margaret P. Manspeaker, Alex Schudel, Lauren F. Sestito, Meghan J. O'Melia, Haydn T. Kissick, Brian P. Pollack, Edmund K. Waller, Susan N. Thomas*

*Corresponding author. Email: susan.thomas@gatech.edu

Published 30 September 2020, *Sci. Transl. Med.* **12**, eaay3575 (2020)
DOI: 10.1126/scitranslmed.aay3575

The PDF file includes:

Materials and Methods
Fig. S1. Changes in CD4 T cell compartment resulting from ICB.
Fig. S2. Therapeutic and staining aPD-1 mAbs simultaneously stain PD-1–expressing T cells.
Fig. S3. mAb fluorescent labeling, accumulation within dLNs, and binding to LN-resident T cells after administration.
Fig. S4. ICB therapy is less effective in larger tumors.
Fig. S5. T_{regs} in TME express CTLA-4 at higher frequencies than helper CD4 and CD8 T cells.
Fig. S6. ICB therapy modulates CD8 T cells in various tissues leading to effector cell phenotypes.
Fig. S7. ICB with vaccination promotes CD4h activation in TdLNs.
Fig. S8. ICB directed to TdLNs alone or in combination with the TME enables dose sparing.
Fig. S9. Effective ICB therapy in breast cancers requires aCTLA-4.
Fig. S10. mAb accumulation in kidneys and lungs proportional to administered dose.

Other Supplementary Material for this manuscript includes the following:

(available at stm.sciencemag.org/cgi/content/full/12/563/eaay3575/DC1)

Data file S1 (Microsoft Excel format). Individual data points for merged or averaged data plots.

Supplementary materials

Materials and Methods

mAb fluorescent labeling

Fluorescent labeling of aCTLA-4 (9H10), aPD-1 (RMP1-14), or aCD3 (KT3) mAb was performed using AlexaFluor647-NHS-Ester (ThermoFisher) for 1 hour, and labeled mAb was purified using a Sepharose CL-6B (Cytiva) column. Labeled mAb fractions were pooled and concentrated using a 10 kDa (Millipore) spin filter. mAb concentrations was determined using a BCA assay (ThermoFisher).

Analysis of non-fluorescent aCTLA-4 accumulation in dLNs

Twenty four hours after i.p. or i.d. administration of 150 μg of aCTLA-4, mice were euthanized and dLNs (axial and brachial) harvested. LNs were placed in optimal cutting temperature (FisherScientific) compound and frozen in 2-methylbutane (SigmaAldrich) solutions using liquid nitrogen. Frozen LNs were sliced using a CryoStar NX70 instrument to 8-10 micrometers. LN sections were serum blocked followed by staining using an AlexaFluor546 conjugated anti-hamster secondary antibody (Abcam). LNs sections were then imaged on a Laser Scanning Confocal microscope (Zeiss 700) and processed using Zeiss ZEN Black 2.3 SP1 software.

Tissue and single cell preparations

After collection, tissues were processed by cutting up to circumvent the possibility of cell enzymatic surface receptor degradation. Single cell suspensions were generated by disrupting tissues through a 70-μm cell strainer (FisherScientific) using a 1 mL syringe plunger followed by two washes in 20 mL of 1X PBS prior to centrifugation and decanting. Red blood cells were lysed with lysing buffer hybrid-max (Sigma) for 7 min at room temperature followed by quenching in PBS. For ex vivo T cell restimulation in Iscove's modified Dulbecco's medium (ThermoFisher) supplemented with 10% FBS (VWR) and 1% penicillin/streptomycin (VWR) (complete medium) or surface/intracellular staining in PBS, either 30% or 70% of total LN cells, respectively, 2 x 10^6 splenocytes, or 5 x 10^6 tumor cells were plated in 96-well U bottom plates (FisherScientific).

Flow cytometry and antibodies

Single cell suspensions from tumors, LNs, and spleens were prepared and after washing, live/dead staining was performed using Zombie Aqua fixable viability kit (Biolegend), followed by wash steps and surface staining with antibodies from BioLegend against the following proteins: CD45 (clone: 30-F11), CD3 (clone: 17A2), CD4 (GK1.5.), CD8 (clone: 53-6.7), PD-1 (clone: 29F.1A12), FoxP3 (clone: MF-14), Tcf1 (clone: S33-966), Tim3 (clone: RMT3-23), Ki-67 (clone: 16A8) Granzyme B (clone: GB11), CXCR5 (clone: L138D7), CD39 (clone: Duha59), IFN-γ (clone: XMG1.2), and TNF-α (clone: MP6-XT22). Surface staining was carried out on ice for 30 min, with tetramer staining done for 15 min. Intracellular staining was performed using the FoxP3 staining kit (eBioscience) according to the manufacturer's instructions. All flow cytometric analyses were performed using a Fortessa flow cytometer (BD Biosciences) and analyzed using Flowjo software (Tree Star).

PD-1 staining with multiple clones

Complete medium was spiked with PMA (SigmaAldrich) and ionomycin (SigmaAldrich), and splenocytes were cultured in this medium for 6 hours. After activation, staining was performed with a surface stain of BV785 labeled aPD-1 (Biolegend, clone: 29F.1A12) or incubated with non-fluorescent aPD-1 (BioXCell, clone: RMP1-14, same clone used in therapeutic experiments) for 20 min on ice, followed by a wash step and incubation with BV785-aPD-1 (Biolegend, clone: 29F.1A12) stain.

Ex vivo T cell stimulation

Complete medium was spiked with either ovalbumin (10 μg/mL, SigmaAldrich) or SIINFEKL (1 μg/mL, SigmaAldrich) and lymphocytes or splenocytes were cultured for 3 hours at 37°C followed by the addition of brefeldin A (1X: BioLegend) for 3 hours. Cells were harvested, stained, and analyzed by flow cytometry as described above.

Alanine transaminase assay

For non-vaccination experiments, mice were treated with aCTLA-4 (9H10) and aPD-1 mAb (150 μg each) on days 5, 7, and 9 for B16F10 experiments, and serum was collected on day 12. For E0771 experiments, mice were treated with aCTLA-4 (9H10) and aPD-1 mAb (100 μg each) on days 10, 14, and 20, and serum collected on day 22. For B16F10-OVA vaccination experiments, serum was collected on day 16. Alanine transaminase activity was measured according to manufacturer's protocol (BioVision).

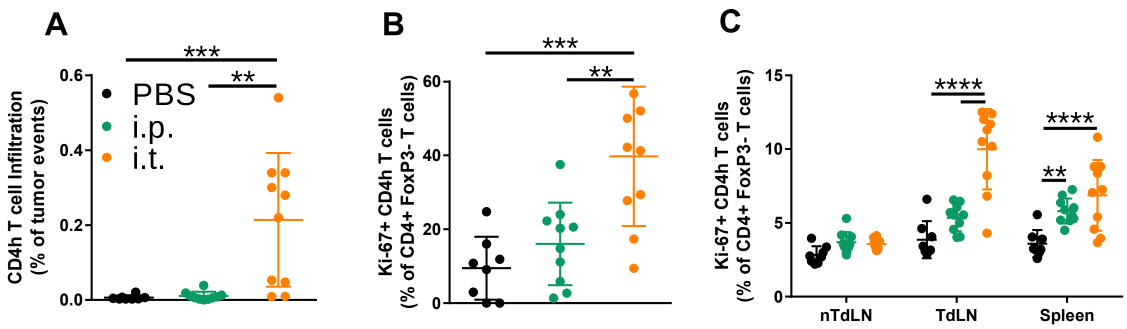

Fig. S1. Changes in CD4 T cell compartment resulting from ICB. (**A**) Frequencies of CD4h (CD4+ FoxP3-) TILs. (**B**) Frequencies of Ki-67+ CD4+ FoxP3- TILs. (**C**) Frequencies of Ki-67+ CD4+ FoxP3- T cells in lymphoid tissues. Data represent two independent experiments (n=8-10 mice), n.s., not significant relative to each group in each tissue. Statistical analyses were done using ANOVA with Tukey's test. *p < 0.05, **p < 0.01, ***p < 0.001, **** p < 0.0001. Data are represented by means ± SD.

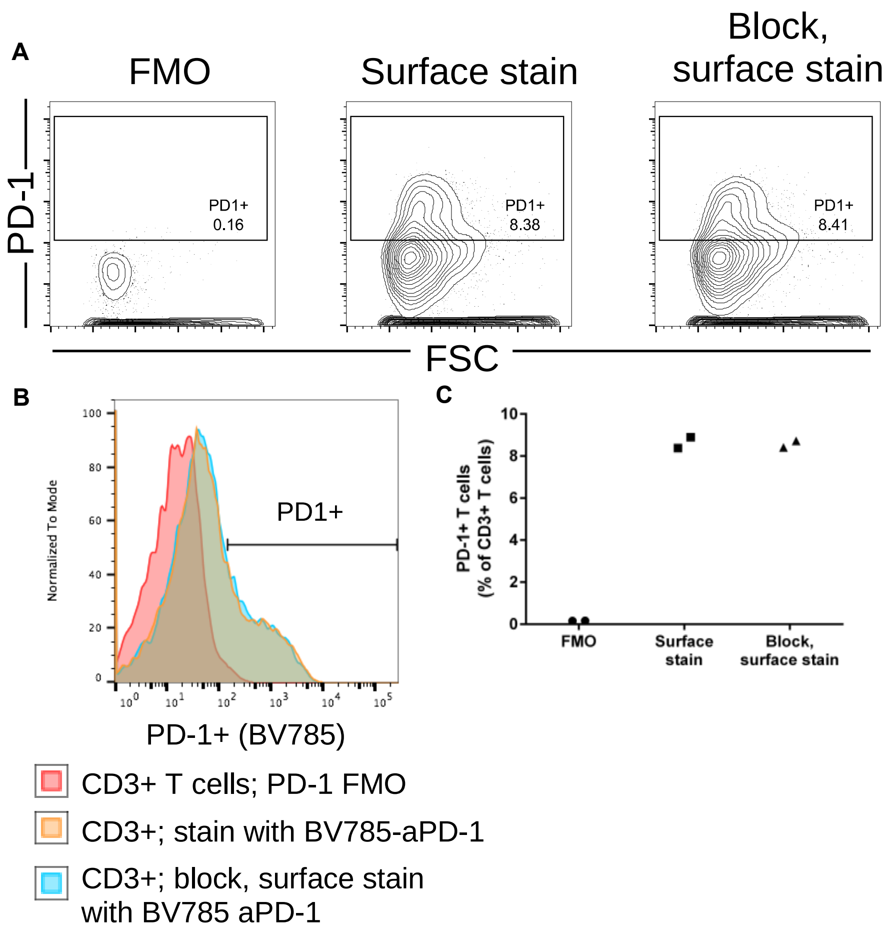

Fig. S2. Therapeutic and staining aPD-1 mAbs simultaneously stain PD-1–expressing T cells. (**A**) Representative flow cytometry plots of splenocytes from each staining group gated on live cells. (**B**) Histogram and quantification of PD-1 expression on CD3+ T cells following activation with ionomycin+PMA using no stain (FMO), BV785 labeled aPD-1 (Biolegend, clone: 29F.1A12) stain, or incubation with non-fluorescent aPD-1 (BioXCell, clone: RMP1-14) followed by BV785-aPD-1 (Biolegend, clone: 29F.1A12) stain. (**C**) Quantification of B. Data represent staining performed on splenocytes from one C57Bl6 animal.

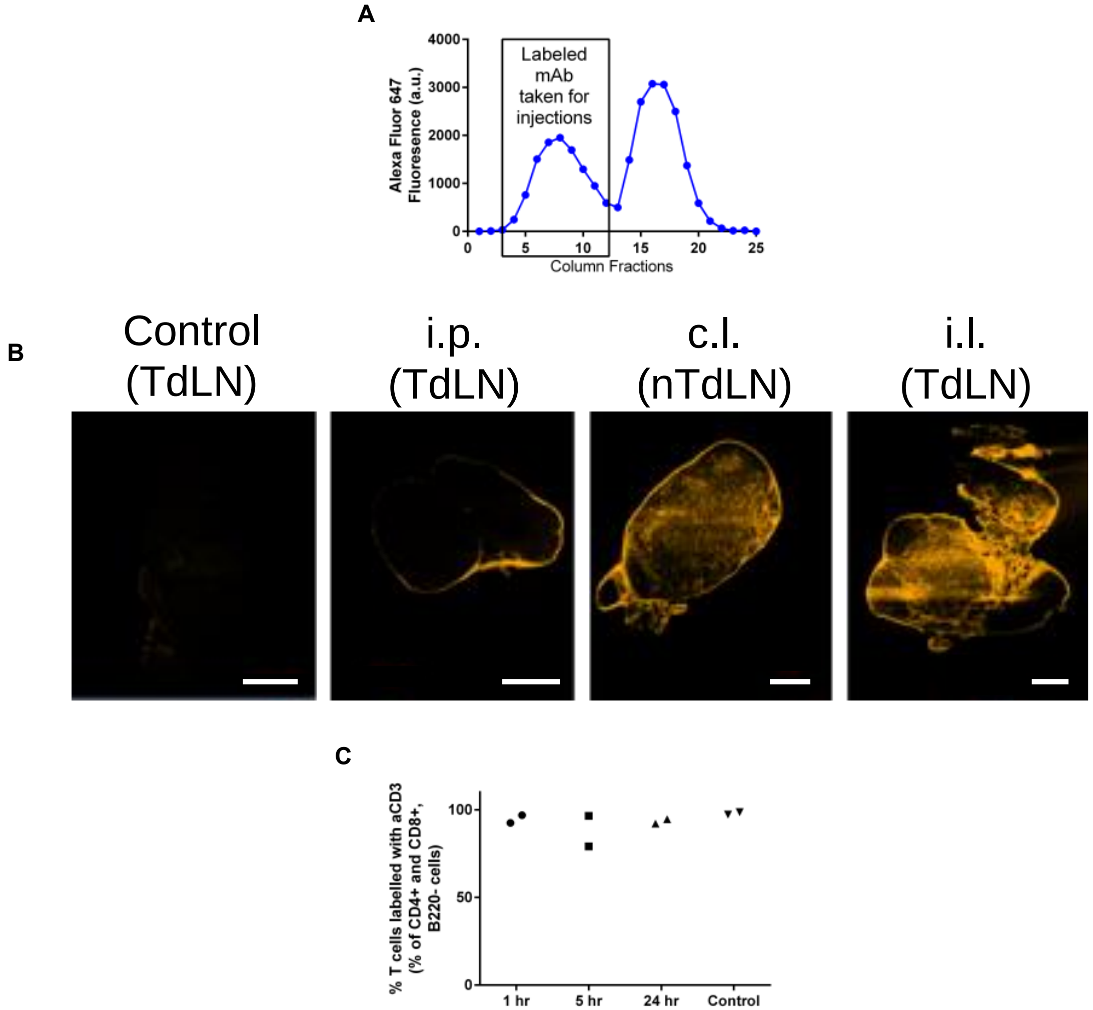

Fig. S3. mAb fluorescent labeling, accumulation within dLNs, and binding to LN-resident T cells after administration. (**A**) Representative size exclusion chromatography curve of Alexa Fluor 647 labeled antibody elution from Sepharose CL-6B. Box indicates collected fractions that were pooled and concentrated for in vivo use. (**B**) Unlabeled aCTLA-4 (9H10) mAb within dLN 24 hr post injection stained ex vivo with fluorescently conjugated secondary antibody (TdLN from uninjected animals as control, TdLN from i.p. or i.l injected animal, nTdLN from c.l. injected animal). Scale bar represents 500 µm. (**C**) Quantification of aCD3 binding to CD3 expressing T cells at different times following collagenase D treatment and LN mechanical disruption. Data represent one experiment (total n=2)).

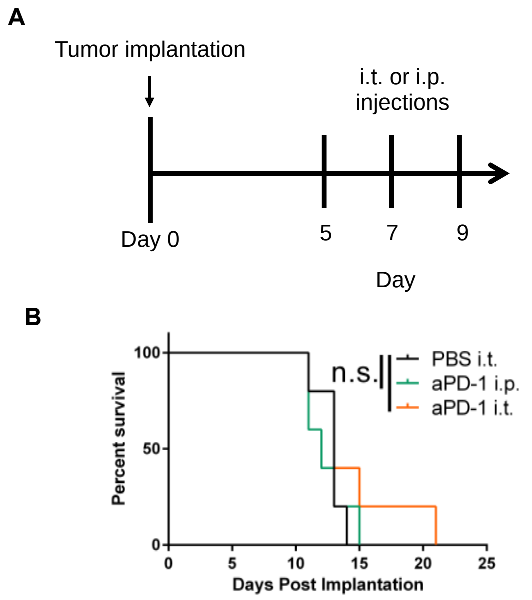

Fig. S4. ICB therapy is less effective in larger tumors. (**A**) C57Bl6 mice were implanted with 5 x 10^5 B16F10 cells on d 0 and treated on d 5, 7, and 9 mice with 150 µg of aPD-1 mAb i.p. or i.t.. (**B**) Survival curves. Data represent one experimental test (total n=5). Log-rank (Mantel-Cox) test for survival curves. n.s. not significant.

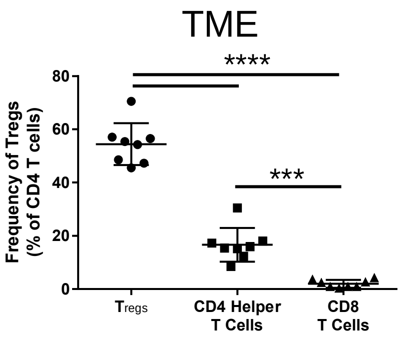

Fig. S5. T$_{regs}$ in TME express CTLA-4 at higher frequencies than helper CD4 and CD8 T cells. 16 d following B16F10-OVA implantation and treatment, mice were euthanized and tumor-resident T cells were analyzed for CTLA-4 expression. Tregs defined as CD4+ FoxP3+ and CD4h T cells as CD4+ FoxP3-. Data represent one experiment (total n=8). Statistical analyses were done using ANOVA with Tukey's test. ***p < 0.001, ****p < 0.0001. Data are represented by means ± SD.

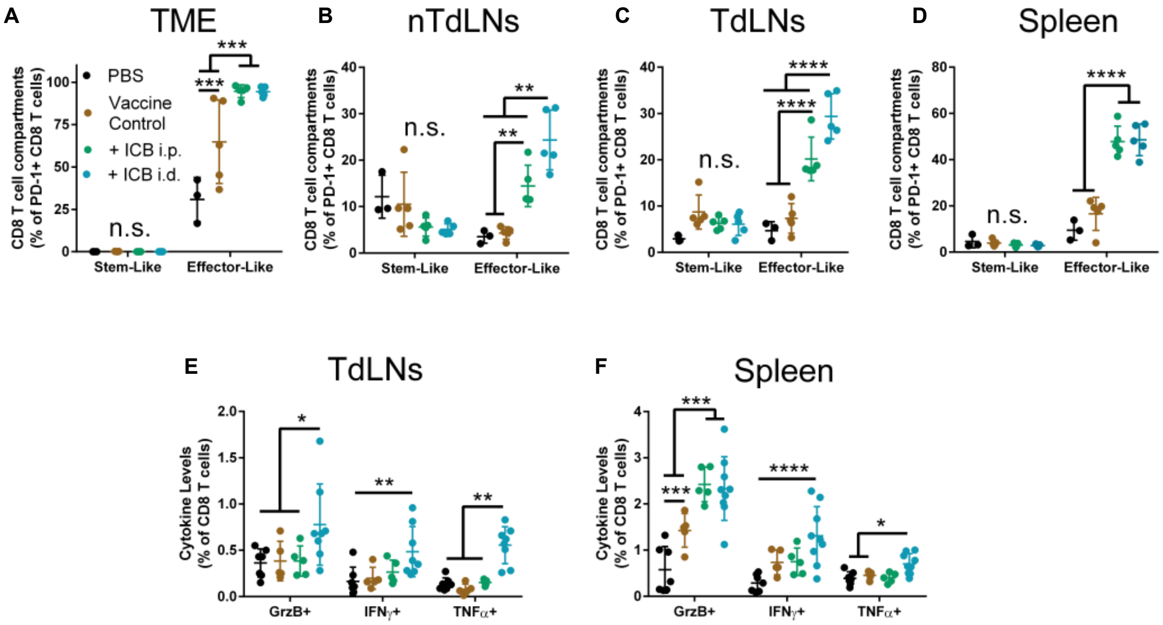

Fig. S6. ICB therapy modulates CD8 T cells in various tissues leading to effector cell phenotypes. 16 d following B16F10-OVA implantation, mice were euthanized and CD8 T cells were analyzed for "stem-like" (PD-1+ CXCR5+ CD39-) or "effector-like" (PD-1+ CXCR5- CD39+) phenotypes. (**A**) TME. (**B**) nTdLNs. (**C**) TdLNs. (**D**) Spleen. E-F) Frequency of effector molecule producing SIINFEKL specific CD8 T cells in TdLNs (**E**) and spleen (**F**). A-D represent one experiment (n=3-5). E-F represent two independent experiments (n=8-10). Statistical analyses were done using ANOVA with Tukey's test. * represents significance, *p < 0.05, **p < 0.01, ***p < 0.001, ****p < 0.0001. Data are represented by means ± SD.

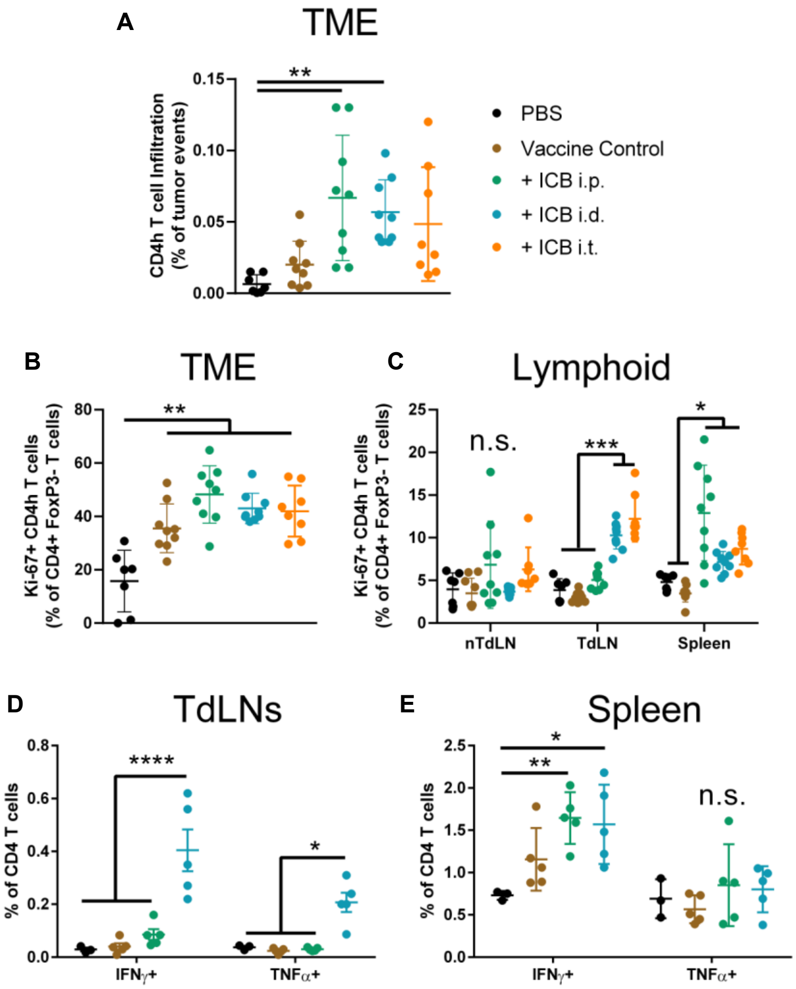

Fig. S7. ICB with vaccination promotes CD4h activation in TdLNs. (**A**) Frequencies of CD4+ FoxP3- T cells in the TME. Frequencies of Ki-67+ CD4+ FoxP3- T cells in TME (**B**) and lymphoid tissues (**C**). D-E) Frequency of cytokine producing OVA specific CD4 T cells in spleen (**D**) and TdLNs (**E**). Data represent one or two independent experiments (n=4-10 mice). Statistical analyses were done using ANOVA with Tukey's test. *p < 0.05, **p < 0.01, ***p < 0.001, ****p < 0.0001, n.s., not significant. Data are represented by means ± SD.

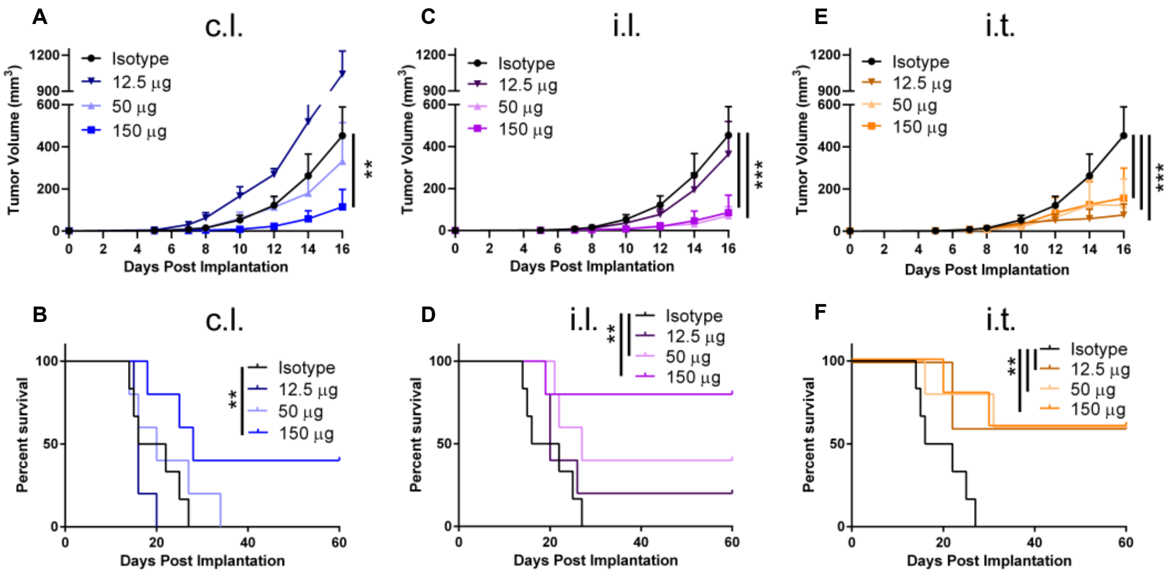

Fig. S8. ICB directed to TdLNs alone or in combination with the TME enables dose sparing. B16F10 tumor growth (A,C,E) and animal survival (B,D,F) using aPD-1+aCTLA-4 (9H10) mAb therapy at various doses; (**A-B**) c.l. administration, (**C-D**) i.l. administration, (**E-F**) i.t. administration. Data represent one experiment (total n=5, mean + SEM). Statistical analyses were done using ANOVA with Tukey's test. Log-rank (Mantel-Cox) test for survival curves. **p < 0.01, ***p < 0.001. Data are represented by means +SEM.

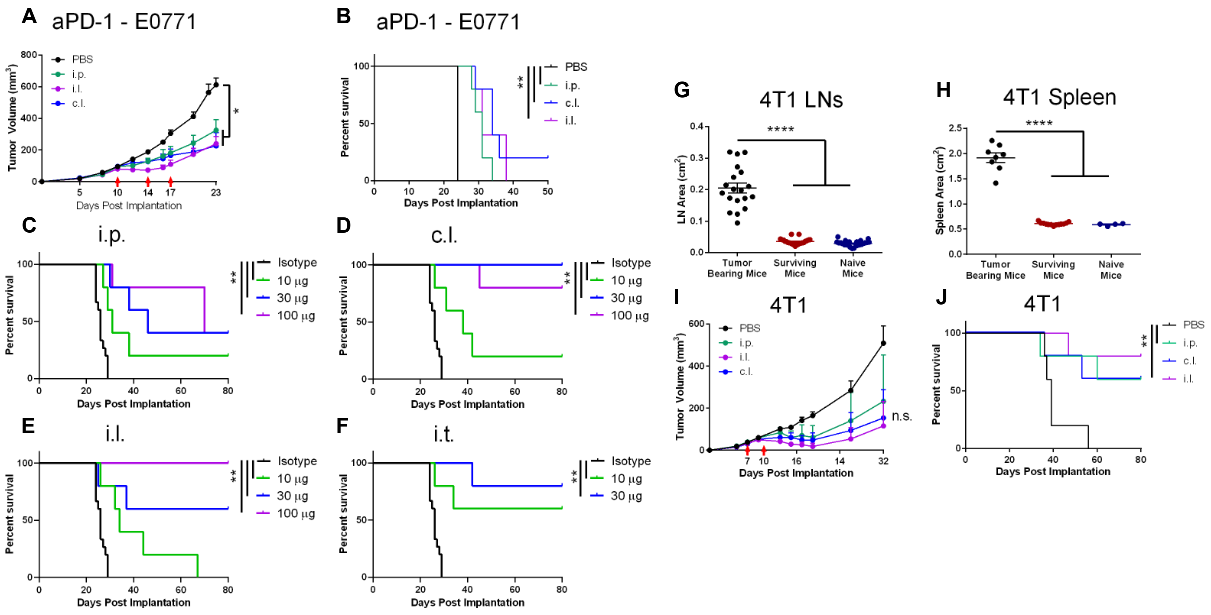

Fig. S9. Effective ICB therapy in breast cancers requires aCTLA-4. (**A**) Growth curves of E0771 tumors with 100 μg aPD-1 monotherapy on d 10, 14, and 17. (B) Survival curves of aPD-1 monotherapy. C-F) Survival curves of E0771 tumors according to administration route with 10, 30, or 100 μg aPD-1+aCTLA-4 (clone: 9H10) therapy starting when tumors reached 100 mm^3 (d 10 or 12) and continued on d 14 and 20 or 16 and 22 after start date: (**C**) i.p. administration, (**D**) c.l. administration, (**E**) i.l. administration, (**F**) i.t. administration. (**G**) Size of LNs at endpoint for tumor bearing mice and d 80 for surviving mice. (**H**) Size of LNs at endpoint for tumor bearing mice and d 80 for surviving mice. (**I**) Growth curve of 4T1 tumors using 50 μg aPD-1+aCTLA-4 (9H10) on d 7 and 10. (**J**) Survival curve of 4T1 mice. Data represent one experiment (total n=4-5, mean ± SEM). Statistical analyses were done using ANOVA with Tukey's test. Log-rank (Mantel-Cox) test for survival curves. *p < 0.05, **p < 0.01, ****p < 0.0001, n.s., not significant. Data are represented by means +SEM (A,I) or ± SD (G-H).

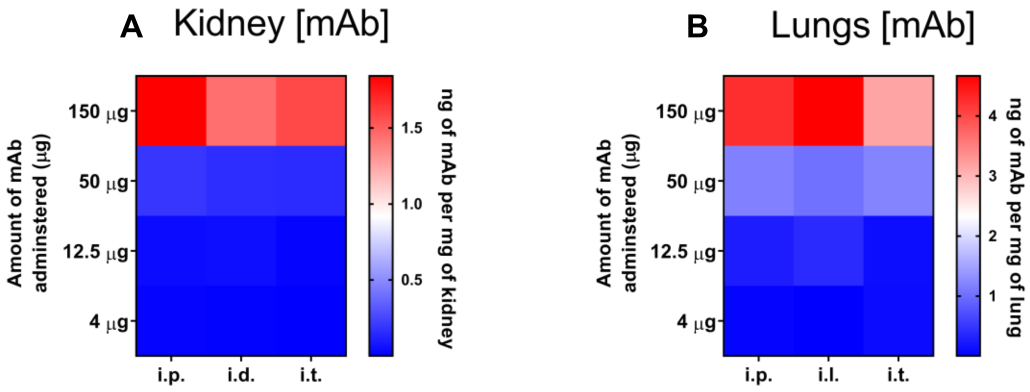

Fig. S10. mAb accumulation in kidneys and lungs proportional to administered dose. 24 hours after administration of aPD-1, mice were euthanized and tissues harvested to measure concentrations of accumulated mAb. (**A**) Kidneys. (**B**) Lungs. Data represent one experiment (total n=2).
